# A Comprehensive Review of LiDAR Applications in Crop Management for Precision Agriculture

**DOI:** 10.3390/s24165409

**Published:** 2024-08-21

**Authors:** Sheikh Muhammad Farhan, Jianjun Yin, Zhijian Chen, Muhammad Sohail Memon

**Affiliations:** School of Agricultural Engineering, Jiangsu University, Zhenjiang 212013, China; farhansheikh@ujs.edu.cn (S.M.F.); 2112116001@stmail.ujs.edu.cn (Z.C.); msohail@ujs.edu.cn (M.S.M.)

**Keywords:** precision agriculture, LiDAR technology, crop management, disease detection, yield estimation, autonomous harvesting systems

## Abstract

Precision agriculture has revolutionized crop management and agricultural production, with LiDAR technology attracting significant interest among various technological advancements. This extensive review examines the various applications of LiDAR in precision agriculture, with a particular emphasis on its function in crop cultivation and harvests. The introduction provides an overview of precision agriculture, highlighting the need for effective agricultural management and the growing significance of LiDAR technology. The prospective advantages of LiDAR for increasing productivity, optimizing resource utilization, managing crop diseases and pesticides, and reducing environmental impact are discussed. The introduction comprehensively covers LiDAR technology in precision agriculture, detailing airborne, terrestrial, and mobile systems along with their specialized applications in the field. After that, the paper reviews the several uses of LiDAR in agricultural cultivation, including crop growth and yield estimate, disease detection, weed control, and plant health evaluation. The use of LiDAR for soil analysis and management, including soil mapping and categorization and the measurement of moisture content and nutrient levels, is reviewed. Additionally, the article examines how LiDAR is used for harvesting crops, including its use in autonomous harvesting systems, post-harvest quality evaluation, and the prediction of crop maturity and yield. Future perspectives, emergent trends, and innovative developments in LiDAR technology for precision agriculture are discussed, along with the critical challenges and research gaps that must be filled. The review concludes by emphasizing potential solutions and future directions for maximizing LiDAR’s potential in precision agriculture. This in-depth review of the uses of LiDAR gives helpful insights for academics, practitioners, and stakeholders interested in using this technology for effective and environmentally friendly crop management, which will eventually contribute to the development of precision agricultural methods.

## 1. Introduction

Precision agriculture is a developing area that uses modern technologies and data-driven methods to maximize crop yield [1,2]. Traditional agricultural practices have drawbacks for productivity, resource use, and crop management [3]. To address these challenges, the integration of LiDAR (light detection and ranging) technology in precision agriculture has emerged as a promising solution. Using laser pulses, LiDAR can produce precise 3D models of the surrounding area for use in remote sensing applications. Since its creation for mapping purposes, LiDAR has found extensive use in several sectors, including forestry, urban design, and, most recently, agriculture [4,5,6]. It is an important tool for crop management since it can record precise, high-resolution data. The introduction of LiDAR technology in precision agriculture has changed crop management techniques, allowing farmers to make educated choices based on accurate and real-time data [7]. LiDAR technology facilitates more targeted interventions and optimizes resource use—such as water, fertilizers, and pesticides—by providing precise data on crop, soil, and field conditions. Agricultural robots equipped with LiDAR enhance various functions, including crop monitoring, disease detection, weed management, yield estimation, mapping, autonomous navigation, and harvesting operations [8,9,10,11]. Several studies suggest combining data from aerial and terrestrial LiDAR systems for crop, canopy, and ground may be a simple, effective, and inexpensive way to enhance site-specific monitoring in agriculture [12,13,14]. There is substantial potential for the use of LiDAR in precision agriculture for crop management. However, this field still requires a thorough examination. LiDAR applications’ present condition may be better understood by researching case studies, reviewing the existing literature, and critically evaluating recent developments.

Previous literature reviews [15,16,17] on LiDAR in precision agriculture have provided valuable insights but have not focused on crop management, particularly crop cultivation and harvesting. Debnath et al. [15] explored LiDAR applications within a limited scope, and Rivera et al. [16] focused exclusively on studies limited to a 5-year timeframe (2017–2022). This comprehensive review examined the application of LiDAR in crop management, emphasizing its role in improving cultivation and harvesting, earlier foundational work, and incorporating the latest advancements. This broader perspective allows us to provide a thorough and up-to-date overview of the possible advantages and difficulties connected with the application of LiDAR technology in crop management methods by concentrating on the unique function of LiDAR technology in these important areas of precision agriculture.

The review paper follows a well-organized structure to explore the critical evaluation of applications of LiDAR for cultivating and harvesting crops in precision agriculture. The paper begins with an introduction that establishes the background and context of precision agriculture, emphasizing the importance of efficient crop management. It then introduces LiDAR technology as an emerging tool with revolutionary potential in this field. The research objective of the paper is to provide a comprehensive evaluation of LiDAR applications. The subsequent sections are divided into key themes. The first section provides an overview of LiDAR technology in precision agriculture, discussing its definition, principles, and different types of LiDAR systems. A key summary of the study results on airborne LiDAR systems (ALSs), terrestrial LiDAR systems (TLSs), and mobile LiDAR systems (MLSs), has been presented in the tables for better understanding and clarity. Key differences between and uses of each LiDAR system are concisely summarized in these tables, which serve as crucial references. The following sections delve into the applications of LiDAR in crop cultivation and harvesting separately, examining its role in crop monitoring, weed detection, plant health assessment, soil analysis, yield estimation, and autonomous harvesting systems. A critical evaluation section follows, exploring the advantages and limitations of LiDAR technology. Various LiDAR systems and their uses in crop management are summarized in detail at the end of the paper. This table provides a brief overview of the cutting-edge technologies being used by scientists all around the globe to improve crop management. It concludes with a comparison of different LiDAR system types (ALS, TLS, and MLS) and their limitations. The paper concludes with future perspectives and challenges, highlighting emerging trends, research gaps, and potential solutions. By following this structured approach, the review paper aims to give a thorough and insightful analysis of the role of LiDAR in revolutionizing crop management in precision agriculture.

### 1.1. Overview of Precision Agriculture

Field-level management techniques for supporting sustainable food production systems are improved by precision agriculture. Furthermore, to produce food sustainably, agricultural operations must be more closely matched to the potential of soil fertility, crop requirements, and environmental circumstances [18]. Precision agriculture aims to maximize agricultural earnings using many essential tactics. It first emphasizes effective resource management by operating systems for applying fertilizers, agrochemicals, and water at varying rates. Ensuring these inputs are dispersed precisely where required helps reduce waste and increase efficacy [19].

Furthermore, precision farming tries to reduce agricultural output losses during harvest. Farmers may decide the best time to harvest their crops and ensure the highest yield and quality using cutting-edge technology and real-time data analysis [20,21]. This strategy strives to reduce the negative environmental impacts of farming. Precision agriculture minimizes fertilizer loss into water bodies and greenhouse gas emissions using focused irrigation and precise input application that protects the environment [22,23]. Finally, precision agriculture aims to minimize the total environmental impact of agricultural inputs. Promoting techniques that support sustainable agricultural systems and boost long-term soil health includes techniques such as carbon sequestration and soil organic matter improvement [24,25]. In conclusion, precision agriculture refers to a variety of techniques that enhance resource management, reduce yield losses, reduce environmental hazards, and maximize the overall effect of agricultural inputs, eventually resulting in higher agricultural profitability and sustainability.

### 1.2. Importance of Efficient Crop Management and the Emerging Role of LiDAR Technology

Effective crop management is crucial in the context of sustainable agriculture and the need to meet the expanding requirements of a rapidly expanding global population [26]. Since LiDAR technologies have the potential to revolutionize precision farming, their development and application in this context have received a lot of attention. The study conducted by Xu et al. [27] research centered on the advancement and assessment of a UAV-LiDAR system designed to facilitate precision agriculture and plant phenotyping. The highest canopy height was estimated with a reported error of 0.1 m. The results provide empirical support for the effectiveness of the LiDAR system, suggesting its potential use in fields including precision crop management and plant breeding. Since LiDAR can take exact three-dimensional data on crop health, terrain, crop breeding, and vegetation structure, farmers may use this information to manage their fields better regarding irrigation, fertilization, insect control, and more [28]. Zhang et al. [29] reported that LiDAR data combined with sophisticated analytics and machine learning algorithms further enhanced capability, enabling farmers to recognize crop-stress zones, adopt better planting practices, and precisely target treatments. LiDAR technology enhances environmental sustainability, reduces resource waste, and boosts agricultural productivity. It aids in creating high-resolution elevation models and vegetation maps, supporting watershed management, land use planning, and climate adaptation while optimizing resource use and advancing sustainable food production amidst growing global populations. It is of the highest significance in modern agriculture to have effective crop management to fulfill the ever-increasing needs for food production while simultaneously reducing the number of resources used and the negative impact on the environment [30,31]. The implementation of precision agricultural methods, such as LiDAR applications, is essential for reaching these goals.

#### 1.2.1. Enhancing Productivity

Using effective crop management techniques, farmers may maximize production potential by carefully matching inputs such as water, fertilizer, and pesticides to the demands of various crops. By delivering thorough and reliable data regarding crop health, growth patterns, and fertilizer needs, LiDAR technology significantly increases agricultural yield. El-Naggar et al. [32] used terrestrial LiDAR for the estimation of crop growth and water use. When compared to manually observed canopy height, the TLS findings showed a noteworthy and statistically significant correlation with minor biases and errors. The R^2^ values of the correlation coefficients for barley, pea, and bean were 5.85 (RMSE = 0.95), 3.01 (RMSE = 0.93), and 1.82 (RMSE = 1.82), respectively. Additionally, the TLS approach showed promise—with an RMSE of 37.56 and an R^2^ value of 0.70 for predicting bean biomass. LiDAR allows farmers to assess crop attributes such as height, density, and canopy structure precisely, using laser beams to produce high-resolution 3D reconstructions of the terrain. According to a study by Eyre et al. [33] geographically weighted regression (GWR) models that utilize topographic variables derived from LiDAR data are highly efficient at identifying field-scale variations in crop yield across various varieties. The coefficient of determination values for maize, wheat, soybeans, and the overall average of all crops were R^2^ = 0.80, 0.73, 0.71, and 0.75, respectively, based on the mean of the local relationships. LiDAR data identifies nutrient deficits and insect infestations, enabling targeted actions. Canopy models provide insights into light, airflow, and shade patterns, optimizing planting densities, irrigation, and pruning.

LiDAR data enhances irrigation design and water management, provides accurate yield estimates, and integrates with remote sensing and analytics for harvest planning and supply chain management. Farmers may embrace precision agriculture, maximize resource allocation, reduce environmental impact, and contribute to efficient and sustainable food production using LiDAR technology with other instruments [34].

#### 1.2.2. Resource Optimization

Precision agriculture has been transformed by LiDAR technology, which has great promise for increasing agricultural output while solving issues with resource efficiency, water shortages, and environmental sustainability. LiDAR provides precise irrigation procedures that save water use while assuring optimum plant development by detecting soil moisture levels and crop water needs [35]. LiDAR is also essential for the targeted administration of pesticides and fertilizers since it helps avoid over-application and possible environmental damage [36]. Farmers can detect regions of changing crop density and health thanks to its capability to build comprehensive 3D models of crop canopies, allowing the creation of prescription maps for exact input applications. This technique, known as variable rate application, minimizes the effect on the environment, uses fewer chemicals, and best utilizes available resources.

Additionally, LiDAR data may be combined with data analytics and machine learning to create predictive models, allowing data-driven decision-making for picking the best crop types, modifying planting densities, and implementing timely interventions [37]. The advantages of the technology also extend to the management of orchards and vineyards, enabling the precise assessments of tree and vine structures for improved pruning techniques, canopy management, and yield estimates. Farmers may achieve sustainable and effective crop production via precision farming techniques by using the power of LiDAR and combining it with other technologies, eventually resulting in a more resource-efficient and ecologically conscientious agricultural sector [38,39].

#### 1.2.3. Disease and Pest Management

The early identification of plant stress indicators and disease signs using LiDAR data helps farmers intervene strategically to slow disease development. Farmers may use LiDAR to administer targeted treatments, eliminating the need to use broad-spectrum pesticides and encouraging sustainable agricultural methods [40]. Incorporating LiDAR data with remote sensing, machine learning, and data analytics enables the creation of predictive models for disease and parasite outbreaks, thereby facilitating proactive decision-making [41]. By giving data on plant water needs and nutrient distribution, LiDAR also helps improve irrigation and fertilization operations. Additionally, the system offers agricultural yield assessment and forecasts, supporting farmers in projecting production potential, optimizing resource allocation, and enhancing harvest procedures. Improved production decreased environmental effects, and the development of sustainable agriculture are all possible because of LiDAR’s capacity to deliver precise crop information, identify plant health concerns, and optimize resource management [42,43].

#### 1.2.4. Sustainability and Environmental Impact

Achieving sustainable agricultural practices is a global priority. Efficient crop management practices supported by LiDAR applications contribute to reducing the environmental footprint of agriculture. By optimizing inputs and minimizing the wastage of resources such as water and fertilizers, LiDAR aids in the reduction of greenhouse gas emissions, nutrient runoff, and soil degradation. This leads to more environmentally friendly and sustainable agricultural systems [44,45].

In conclusion, efficient crop management is crucial for meeting the increasing demand for food production while minimizing resource utilization and environmental impact.

## 2. LiDAR Technology in Precision Agriculture: An Overview

### 2.1. LiDAR in Precision Agriculture

**Figure 1:**
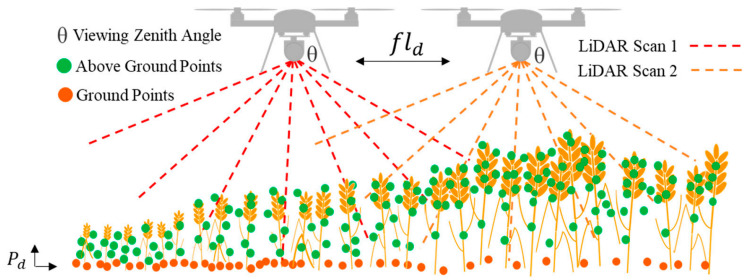
Use of LiDAR in precision agriculture [51].

LiDAR technology operates based on the principles of laser ranging and time-of-flight (TOF) measurement. It involves emitting laser pulses towards the target area, which bounce back when they encounter objects or surfaces, allowing for precise distance calculations [46,47]. LiDAR systems provide precise distance measurements by measuring the time it takes for the laser pulses to return. To create accurate 3D maps of the surroundings, these data are combined with the angles and placements of the laser pulses. LiDAR scanners generate laser beams in a pattern that scans a large region while quickly capturing several points in each location. These points precisely depict the shape outlines and spatial features of the items in the scanned area, forming point clouds together. Additionally, it is possible to measure the strength of the returned laser pulses, providing extra information on the reflectance or surface characteristics of the objects [48,49,50]. Figure 1 provides a detailed explanation of the LiDAR system’s operating concept as described by Bates et al. [51].

The distance a single photon has traveled to and from an object is calculated using Equation (1):

d = c t/2,(1)

where “d” is the distance from an object; “c” is the speed of light, whose value is constant; and “t” is the time-of-flight, which can be measured by the difference between the start time of the emitted pulse and the time the reflected pulse hits the sensor.

Phase shift measurement (PMS), which uses a continuous light source, modulates light power at a fixed frequency. As a result, we may say that the modulated light has a sinusoidal profile. Equation (2) may be used to calculate the distance between the source and the object in terms of the angle between the waves’ peaks:

d = c∆θ/2πf(2)

where “d” is the distance, “c” is the speed of light, “∆θ” is the phase difference, and “f” is the frequency of the modulated power. TOF or PMS distance measurements are converted into elevation data. These elevation data appropriately represent numerous ground objects in the scanned region. Researchers and professionals can effectively visualize and analyze the terrain, the topography, and the structural characteristics of ground objects by utilizing the precise elevation data derived from TOF or PMS measurements [52].

LiDAR has gained recognition in precision agriculture as a powerful tool for crop monitoring, management, and harvesting due to its ability to capture highly accurate and detailed spatial information. By providing precise measurements of crop height, canopy structure, and vegetation density [53,54], LiDAR enables farmers and agronomists to assess crop health, monitor growth patterns, and detect variations or stress factors [55,56]. Furthermore, LiDAR assists in soil analysis, allowing for precise soil mapping, the identification of soil properties, and the assessment of soil erosion risks. This spatial information empowers farmers to make data-driven decisions for optimizing irrigation, fertilization, pest control, and overall crop management.

### 2.2. Types of LiDAR Systems and Their Use in Precision Agriculture

**Figure 2:**
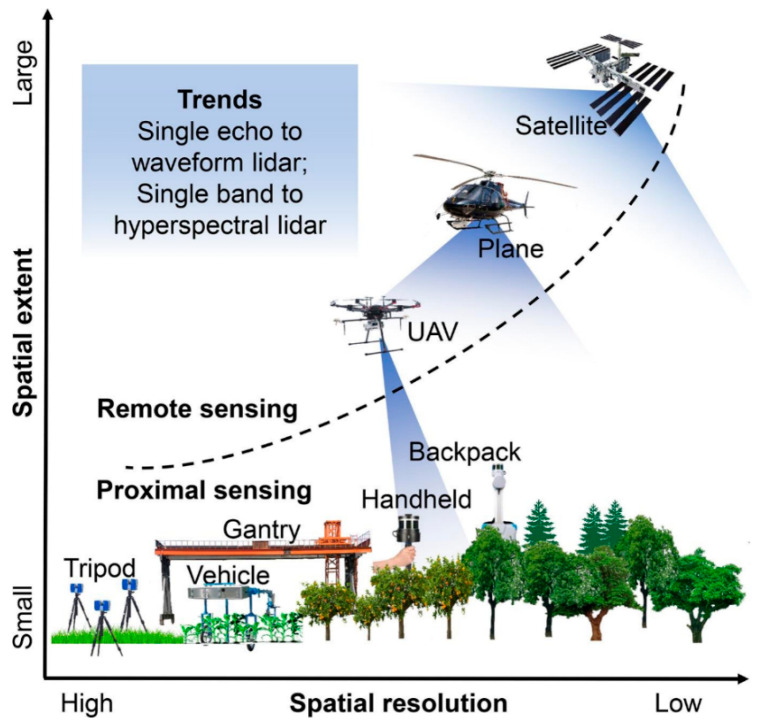
Different types of LiDAR systems are used in precision agriculture [57].

A detailed overview of several LiDAR systems and their specialized applications in precision agriculture is explained by Jin et al. [57], as presented in Figure 2. Researchers categorized LiDAR systems based on the range of LiDAR sensors, distinguishing between proximal sensing and remote sensing. Proximal sensing platforms, such as terrestrial LiDAR systems (TLSs) and mobile LiDAR systems (MLSs), and remote sensing platforms, such as airborne LiDAR systems (ALSs), play distinct roles in precision agriculture, each contributing unique capacities to the field. By analyzing these various LiDAR technologies in-depth, this review provides valuable insights into how each system can be utilized for crop management.

#### 2.2.1. Airborne LiDAR Systems (ALSs)

**Figure 3:**
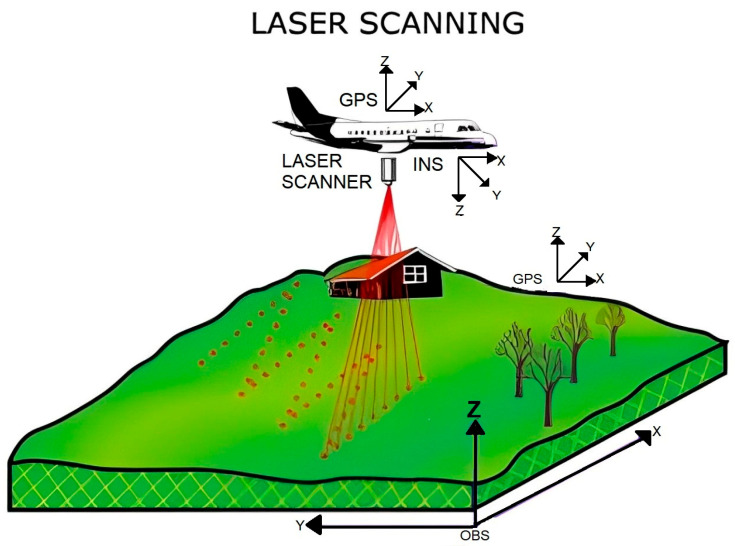
Airborne LiDAR system [58].

**Figure 4:**
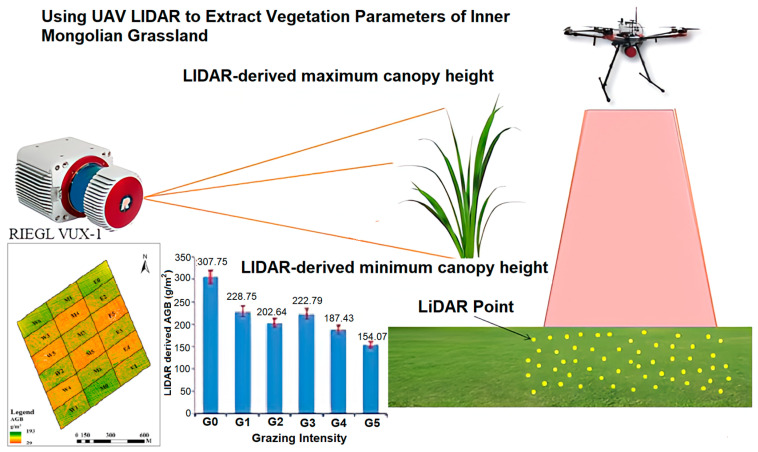
UAV-mounted ALS for canopy height estimation [69].

Airborne LiDAR systems involve the installation of the LiDAR system onto unmanned aerial vehicles (UAVs), which can be aircraft or drones, allowing them to efficiently collect data at low altitudes over agricultural fields, as shown in Figure 3 and Figure 4. These systems emit laser pulses and measure the time it takes for the pulses to return after hitting the target objects [58].

By analyzing the returned signals, the ALS can create highly accurate 3D models of the terrain and vegetation [59]. They provide valuable information for precision agriculture, such as crop health assessment, vegetation mapping, and canopy structure analysis [60]. The ALS provides high-resolution data, offers a cost-effective solution for precision agriculture, and has been successfully used for crop monitoring, terrain modeling, and flood risk assessment in various agricultural settings [61]. Rakesh et al. [62] reported that ALSs can perform targeted analysis and decision-making for crop management, such as crop yield estimation, disease detection, and irrigation management. Fareed et al. [63] stated that ALSs can capture detailed information about crop conditions, plant health, and pest infestations at the field level. After a comprehensive review, Aslan et al. [64] concluded that ALS is particularly useful for field mapping, yield estimation, and resource optimization. Dowling et al. [65] and Qin et al. [66] employed UAV-LiDAR for mapping and navigation and demonstrated effective obstacle avoidance capabilities in their studies. Turner et al. [67] used ALS observations to map soil surface roughness (SR) in agriculture. The findings demonstrated that soil profile surface heights estimated by ALS were more precise and accurate than those estimated by ground measurements. The effects of farming activities on surface roughness were tracked using LiDAR data, which showed promising results for mapping SR in agriculture. Ladefoged et al. [68] found geographical and temporal trends in the evolution of local agricultural systems using a high-resolution ALS. Researchers emphasized that integrating LiDAR data with productivity models improved the comprehension of agricultural growth in the Hawaiian area. Zhang et al. [69] reported that accurate estimation of grassland vegetation parameters such as maximum, minimum, and mean canopy height, AGB, and fractional vegetative coverage at a high spatial resolution can be achieved with a UAV-mounted ALS, as presented in Figure 4.

Liu and Bo [70] used the UAV–LiDAR system to identify different crop species in the complicated, fragmented agricultural landscape and crop planting structure by using canopy height model (CHM) data. The accuracy of crop species classification was significantly improved by including geometric and textural cues in the object-based classification technique. The suggested object-based classification framework enabled the research to categorize crop species with an overall accuracy of 90.33%. Table 1 illustrates the diverse applications of ALSs and important findings from the literature in the realm of crop management. The classification, implementation, and limitations of ALSs are presented in Table 2.

#### 2.2.2. Terrestrial LiDAR Systems (TLSs)

**Figure 5:**
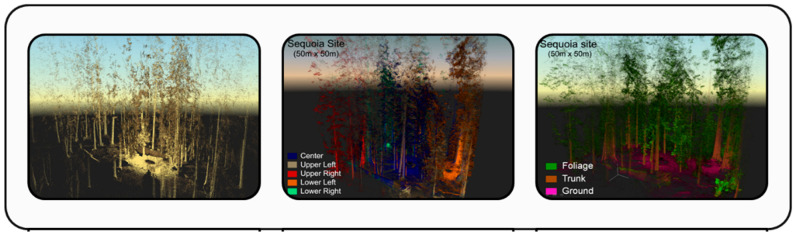
TLS for crop height estimation [81].

TLSs are ground-based setups that utilize stationary tripod stands to scan and capture detailed crop information at different locations. These systems emit laser pulses in various directions, capturing multiple measurements from different angles. By combining these measurements, TLSs generate precise 2D and 3D point cloud data of the surrounding environment, as shown in Figure 5. 

Many researchers reported that TLSs are especially effective for detailed mapping of crop structures, including plant height, canopy density, and individual plant or tree measurements. In contrast to UAV-based LiDAR, which typically covers larger areas from above, a TLS provides high-resolution, ground-level data that offers greater precision in assessing specific crop attributes and intricate details within a given area. It is also reported that TLSs are superior to UAV–LiDAR for accurate crop health assessment, growth pattern assessment, and biomass estimation at the field level. Researchers have utilized TLSs for crop phenotyping, growth analysis, and precision irrigation management [82].

**Figure 6:**
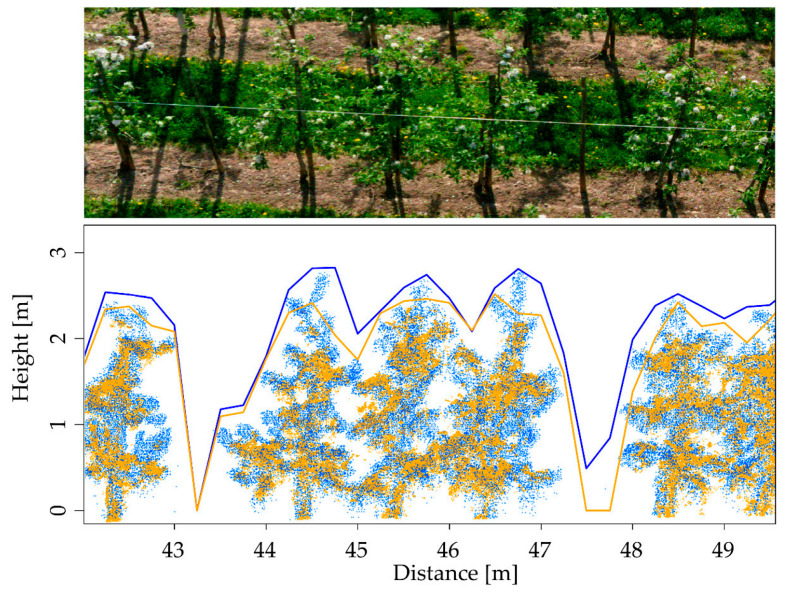
View of apple trees rows as an RGB, point clouds from drones (orange) and LiDAR (blue) superimposed with tree wall height curves [85].

Hosoi and Omasa [83] estimated vertical plant area density profiles of the wheat canopy during several development phases, including tillering, stem elongation, blooming, and ripening, using a portable TLS. By setting up regression models between LiDAR-measured plant area density and organ dry weight, the researchers were able to determine the total dry weight and carbon stocks of above-ground wheat organs. Researchers concluded that these results could help better understand how carbon is stored in agricultural systems and improve crop management techniques and carbon sequestration methods. Martínez et al. [84] used a TLS to measure the LAI of grapevine and reported that the LiDAR sensor can also produce maps if the maximum distance between scan points does not exceed 15 m. However, to avoid LAI overestimation, increasing the horizontal resolution of LiDAR scanning is essential. Hobart et al. [85] used drones and photogrammetry to measure tree wall heights in apple orchards and compared them with TLS data as a reference, as described in Figure 6. Researchers reported that although there was a good match between the drones’ point clouds and the ground-based LiDAR data, the drones’ point clouds had trouble collecting the fine apple tree shoots, which resulted in lower assessments of tree wall heights. The method can be used to manage orchards precisely, but it must be modified to consider the wider tree gaps and the decreased vertical extent of tree walls.

**Figure 7:**
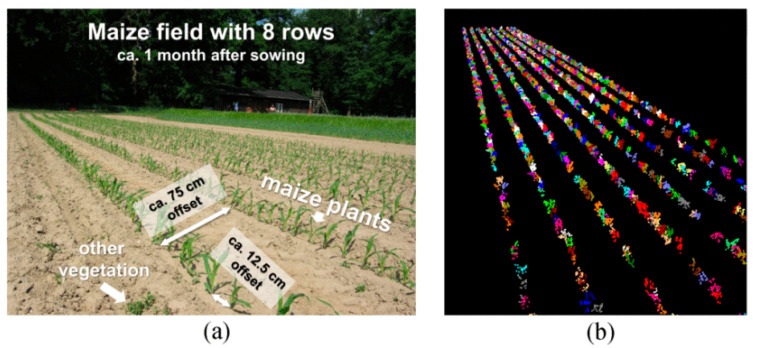
(**a**) Maize plants (arranged in 8 rows) with additional vegetation, and (**b**) a 3D model of the segmented point cloud [86].

Using a TLS, Hofle [86] described a unique method for mapping individual maize plants. The developed method involved point cloud segmentation and filtering detected plants accurately and consistently, as shown in Figure 7. The amplitude variation of homogenous regions was minimized using radiometric correction, improving the separability of maize plants and the surrounding soil with greater accuracy when applied to LiDAR point clouds taken by TLS, which provided benefits including lessened obstruction effects and more uniform point density.

Xu et al. [87] developed a unique precision farming technique combining TLS and camera data to identify corn seedlings in fields precisely. The technique required removing distance effects from TLS intensity data, registering point cloud and camera data for proper color representation, and separating corn plants from the soil with a random forest algorithm employing geometric and radiometric parameters. The performance of employing either feature type alone was improved in a case study using a commercial TLS sensor with an integrated camera, which showed a high accuracy of 98.8% in distinguishing maize seedlings from the soil. The results of Koenig et al. [88] highlight the significant effectiveness of geometric and radiometric LiDAR point cloud characteristics in accurately classifying tree induction data to identify post-harvest growth. Key findings from the literature are included in Table 3, which shows TLS uses in crop management.

#### 2.2.3. Mobile LiDAR Systems (MLSs)

Mobile LiDAR systems are portable devices that allow users to collect data by mounting the scanners on manned or unmanned ground vehicles such as tractors or robot vehicles, which are autonomous or remotely controlled and designed to operate on land. It can even be mounted on a backpack or held by a person walking. These systems are typically lightweight and easy to operate [99]. With the MLS, researchers and farmers can capture localized information and perform detailed measurements in smaller areas or specific field sections. They provide the ability to assess crop height variations, monitor canopy structure, and identify micro-environmental factors that influence crop growth. The MLS is useful for on-the-spot assessments and can complement data from other LiDAR platforms. Researchers have utilized MLSs for crop height estimation, plant architecture analysis, leaf area index, and precision nutrient management [100,101].

**Figure 8:**
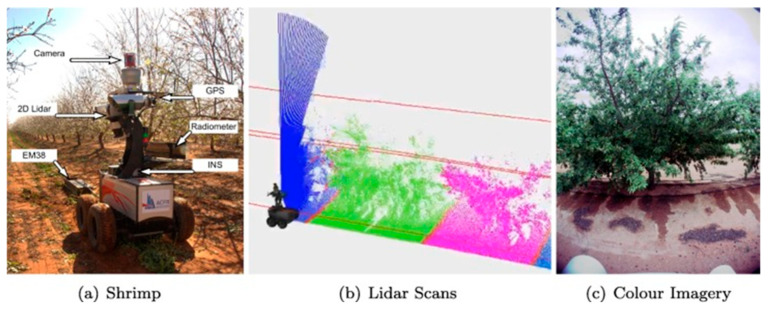
Mapping an almond orchard using an MLS [103].

Zhou et al. [102] reported that MLSs performed better than ALSs when scanning tree growth. Point cloud data provides more precision and completeness than an MLS and can accurately extract forestry characteristics and record individual tree branch structures. Underwood et al. [103] employed an MLS to efficiently map flower and fruit distributions and forecast individual tree production in almond orchards, as depicted in Figure 8. The technology employed a robotic ground vehicle equipped with LiDAR and video sensors to generate a 3D map of the orchard and identify the plants throughout the year. A significant linear relation existed between canopy volume measured by LiDAR and yield. The created maps were used to classify almonds correctly. Researchers concluded that these findings advance precision agriculture by providing valuable insights for optimizing almond orchard management and increasing the accuracy of yield prediction.

Arno et al. [104] predicted LAI by transversely surveying grapevines along the rows using MLS sensors placed on a tractor. Vine height, cross-sectional area, canopy volume, and tree area index (TAI) were calculated. The TAI significantly correlated with LAI values, demonstrating the viability of using LiDAR sensors to characterize grapevine leaves. In the wheat improvement project, Deery et al. [105] assessed the repeatability of nondestructive measurements of AGB and crop growth rate (CGR) using an MLS. The research compared two LiDAR-based techniques for calculating AGB using damage data. Different water supply levels and wheat genotypes were the subjects of several investigations. According to the findings, there are strong correlations between MLS-derived biomass indices and AGB, suggesting that MLSs may serve as a reliable substitute for AGB. The study’s overall findings demonstrate MLSs’ potential as a trustworthy tool for evaluating AGB and CGR in wheat breeding and research.

**Figure 9:**
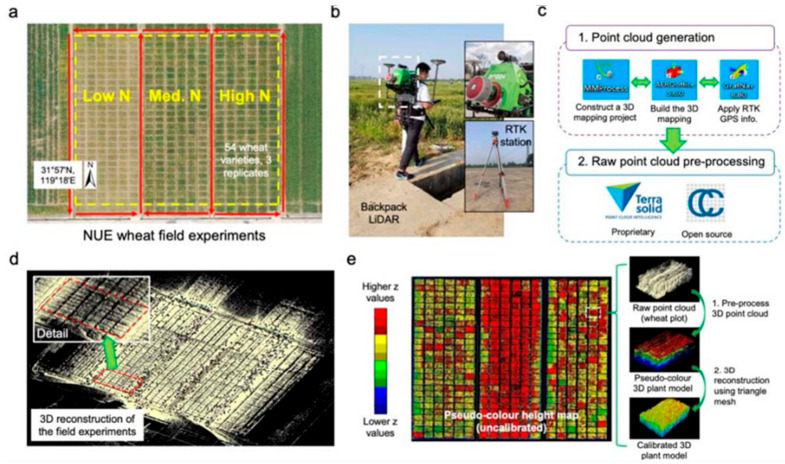
Data acquisition using backpack LiDAR system (**a**) Aerial view of the experimental site (**b**) Backpack LiDAR system (**c**) Function and data acquisition (**d**) 3D reconstruction of the experimental field (**e**) AGB estimation through point clouds data [107].

In their work, Ruhan et al. [106] estimated the aboveground AGB of individual trees using a backpack LiDAR system and optimized quantitative structural models (AdQSM). They were able to accurately determine the diameter at breast height (DBH) of each tree by using the point cloud data obtained from the backpack LiDAR system. The investigation confirmed that the use of backpack LiDAR in a non-destructive manner could produce accurate estimates of the AGB of individual trees. A unique phenotyping approach has revolutionized field-based plant research, as Zhu et al. [107] demonstrated. It combined a backpack LiDAR device with the GUI program Crop Quant-3D, as shown in Figure 9. The method made it possible to collect millions of 3D points to measure crop height and complicated features precisely, such as changes in canopy structure. With good correlations to traditional manual measures, the integrated approach effectively separated genotype and treatment effects on important morphological features. The system showed potential in effectively utilizing current genetic data for improved plant phenomics research, yet there is potential for improvement in accuracy and cost.

Guo et al. [108] developed and tested Crop 3D, a high-throughput crop phenotyping technology. They used LiDAR technology with other advanced sensors to gather phenotypic data from several sources during the crop growth cycle. The researchers discussed the platform’s design, functionality, testing outcomes, and prospective applications and prospects in crop phenotyping. They concluded from their research that systems merging LiDAR and conventional remote sensing methods could be the future of high-throughput crop phenotyping. An overview of the various ways in which the MLS optimizes agricultural practices is presented in Table 4, which provides a concise summary of MLS applications in crop management and highlights significant findings from the relevant literature.

## 3. Applications of LiDAR in Crop Cultivation

### 3.1. Crop Monitoring and Management

Precision agriculture relies on effective crop monitoring and management strategies, and LiDAR technology offers valuable tools for these tasks [117]. By utilizing LiDAR data, farmers and agronomists can gain insights into plant health, disease detection, weed management, and crop growth monitoring [118,119,120].

**Figure 10:**
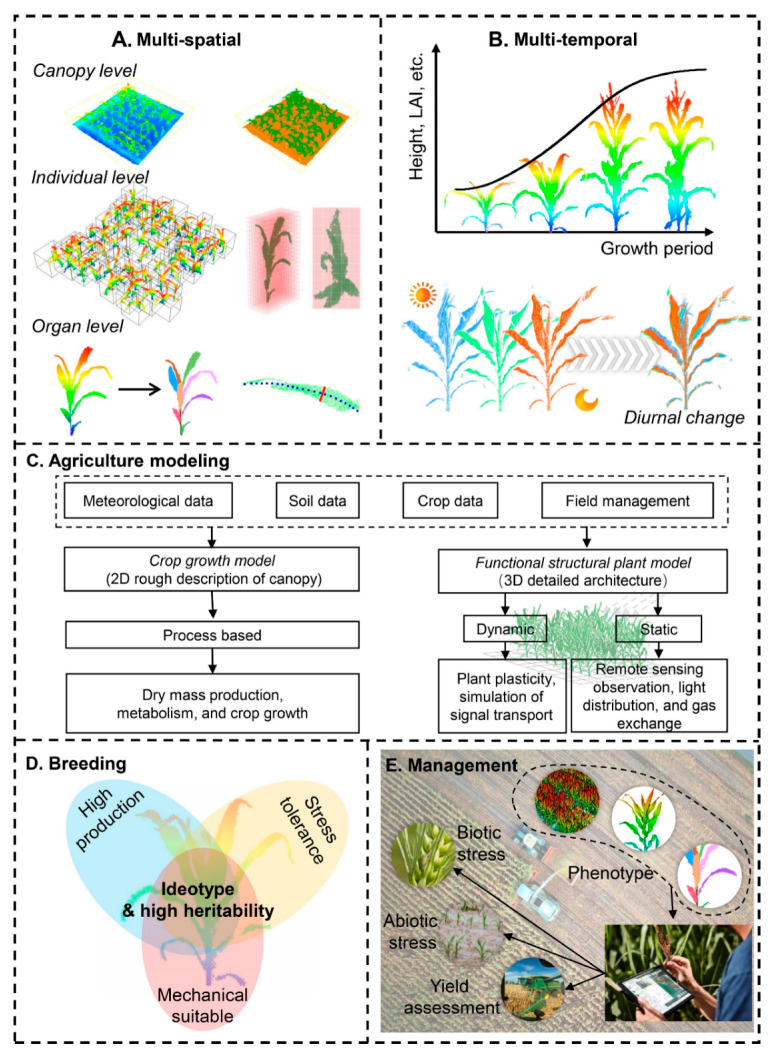
An overview of applications of LiDAR in agriculture (**A**) canopy and individual height measurement, (**B**) growth prediction and measurement, (**C**) field management practices, (**D**) breeding, (**E**) crop management practices) [57].

Figure 10 presents an overview of the applications of LiDAR in agriculture for crop monitoring and management practices, as using just RGB data prevents the correct extraction of plant structural information. LiDAR, a potentially active technique, deals with overcoming this problem by sending laser pulses through tree canopies and picking up vegetation in the understory. LiDAR delivers accurate, three-dimensional (3D) plant data by measuring the return pulse time. LiDAR sensors have been successfully used in several successful projects to estimate different vegetation parameters, including vegetation height [121], coverage [122], leaf area index [123], above-ground biomass [124], and crown size and volume [125]. For example, Brede et al. [126] used the TreeQSM approach to estimate tree volume in their comparison of UAV laser scanning with terrestrial laser scanning. A machine learning–based classification method was carried out by Moorthy et al. [127] to discriminate between woody and leafy components in 3D point clouds. In most instances, the solution outperformed previous methods, eliminating the need for additional post-processing procedures. Based on their research, Montzka et al. [128] concluded that crop height, gap percentage, and intensity are three LiDAR metrics that can predict the AGB, with slightly improved accuracy when testing plants in wet environments. In recent years, LiDAR technology has enabled precise and detailed measurements of crop characteristics [129,130,131,132], such as crop height [133], canopy density, plant growth rate [134], plant species diversity [135], and aboveground fresh and dry biomass [136,137,138]. This information allows farmers to monitor the health and development of crops more accurately. By analyzing LiDAR data, they can identify variations in plant height, which may indicate uneven nutrient distribution or pest infestations [139]. Detailed information regarding these applications is described in Figure 10.

#### 3.1.1. Plant Health Assessment, Maintenance, and Disease Detection

Traditional methods for plant health assessment, maintenance, and disease detection are often unreliable and costly. In contrast, LiDAR technology offers detailed information on vegetation structure, crop growth, leaf area, and canopy density-key indicators of plant health—providing a more accurate and cost-effective solution. By analyzing LiDAR-derived metrics such as plant height, canopy volume, or leaf area index, researchers and farmers can assess the overall condition of crops [140]. Deviations from expected values may indicate stress, nutrient deficiencies, or disease presence. The early detection of diseases is crucial for implementing timely interventions, such as targeted pesticide application or adjusting irrigation and fertilization practices [20].

**Figure 11:**
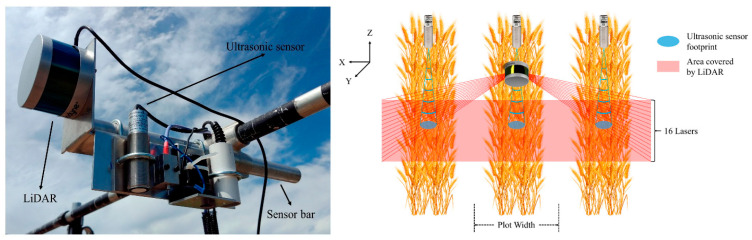
Phenotyping system and scanning areas of LiDAR and ultrasonic sensors [145].

LiDAR technology has been extensively studied for estimation-oriented agricultural applications, including maintenance tasks. It has proven effective across different soil types and temperature conditions, including in orchards, providing valuable insights for crop management and optimization. For example, Escola et al. [141] substitute conventional approaches to calculate the number of trees in an olive grove by using an MLS. This approach was also used by Sandonis-Pozo et al. [142] to estimate the canopy characteristics of almond trees to locate the canopy areas requiring maintenance. Anken et al. [143] used a more accurate method for the precise measurements of canopy area in a project named ‘3D Mosaic’, which aimed to better regulate water and fertilizer use in orchards. Using conventional cameras, two techniques were used in a plum orchard in Potsdam, Germany: LiDAR (plane-mounted laser) and NIR (near-infrared) imaging. Both techniques generated precise canopy size measurements and demonstrated a good connection with real leaf area. The camera system provided a more affordable alternative, while the LiDAR system has the benefit of easy data processing. Moreover, traditional methods of measuring the height of wheat are labor-intensive and prone to inaccuracy. However, since it reveals the yield and weather resilience, this assessment is significant for this crop type. Dhami et al. [144] presented methods for assessing wheat crop heights using a 3D LiDAR sensor mounted on a UAV. They created a mechanism for retrieving plant heights from 3D LiDAR data in plot-based phenotyping contexts. The researchers also created a toolchain for modeling phenotyping farms and determined plant height in a wheat field with an accuracy of RMSE of 6.1 cm. Notably, their method accurately predicted plant heights in a field with 112 plots, making it the first time 3D LiDAR data were gathered over a wheat field by an aerial robot. A multi-sensor phenotyping system was developed by Yuan et al. [145] for estimating wheat canopy heights using ground phenotyping technologies, UAV, and manual measurements. Ultrasonic sensors and LiDAR technologies were used in the system, as shown in Figure 11. The accuracy of wheat height estimates was improved by pre-processing LiDAR data to counteract the slanting effect. The RMSE for LiDAR was 0.05 m, and the R^2^ for the correlation between manual measurements and LiDAR was 0.97. The RMSE for UAV was 0.09 m, and the R^2^ for the correlation between manual and UAV was 0.91. Both measurements were used to determine canopy height. LiDAR produced the most accurate findings, with an R^2^ of 0.97 and an RMSE of 0.05 m.

**Figure 12:**
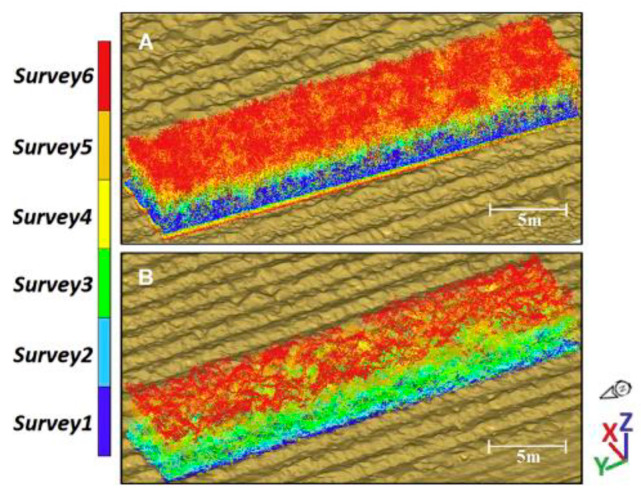
(**A**) LiDAR and (**B**) photogrammetry time-series point cloud data colored by survey (dark blue to red) [149].

Meanwhile, maize heights were examined by Ziliani et al. [146] and Gao et al. [147]. With this metric, one can get an overall assessment of the plant’s health condition. With this index, farmers can evaluate the overall condition of the plant. In related research, Zhou et al. [148] employed an ALS to track maize development and examine how the climate affected this plant over the lodging season. Estimating sugar cane yields requires careful crop observation during its entire development cycle. Sofonia et al. [149] monitored sugar cane yield with different nitrogen fertilizer treatments (0, 70, 110, 150, and 190 Nkg·ha^−1^) in Australia using an ALS. This study aimed to simultaneously compare two systems, CSIRO Hovermap LiDAR, and Micasense RedEdge multispectral camera, to conclude the relationships between plant height, biomass, and yield, as shown in Figure 12. The results indicated that both technologies offer accurate crop height measurements and timely problem detection. However, UAV–LiDAR exhibited greater consistency and stronger correlations when analyzing the biophysical properties of sugarcane compared to optical remotely sensed data.

Ghamkhar et al. [150] suggested employing an MLS to do this effectively. They were the first to get findings that could be put into practice and compared to the conventional method. 

#### 3.1.2. Weed Detection and Fertilizer Application

**Figure 13:**
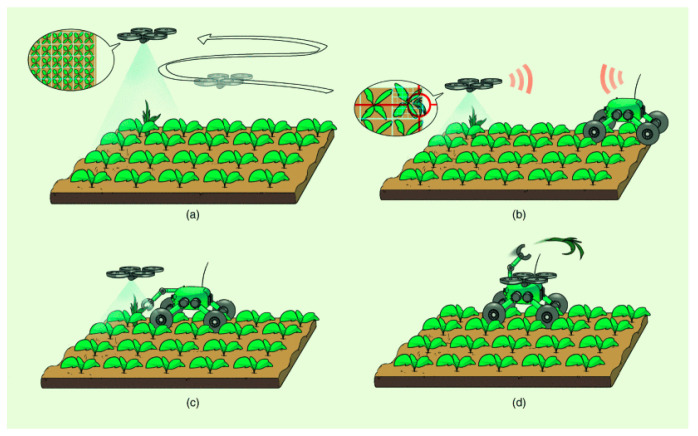
Conceptual identification and removal of wild plants using an ALS and a TLS (**a**) Weed detection via ALS (**b**) Signals transfer to TLS (**c**) Navigation to exact location (**d**) Removal of weeds via TLS [152].

Effective weed detection, control, and precise fertilizer application are vital for optimizing crop productivity. LiDAR technology enhances these practices by offering detailed insights into plant height and canopy structure. This enables accurate weed detection and targeted fertilizer application, as LiDAR can differentiate between crops and weeds based on variations in vegetation height and structure. This enables the development of precise and targeted weed control strategies, reducing the reliance on broad-spectrum herbicides and minimizing the potential negative environmental impact [151]. Removing weeds from fields before they consume crop nutrients is essential. As a result, it is essential to constantly monitor crops for the presence of wild plants and remove them before they may spread disease. Pretto et al. [152] provide an alternate method for the identification of wild plants by combining the use of a TLS and an ALS. The researchers in this study detect and manage the removal of wild plants with the help of an ALS and a TLS, as shown in Figure 13. Computer vision technologies such as LiDAR were used to navigate robotic vehicles during agricultural exploration, and weed detection was accomplished using the AgriColMap method. This vehicle can estimate the density of the crop and use that information to guide itself around the field.

Liu et al. [153] used UAV-LiDAR to estimate differences in the plant height of cotton plants across space. The research discovered a maximum relative error of 12.73% and an error value of 3.48 cm by comparing hand plant height measurements with LiDAR measurements. The coefficient of variation analysis showed height variations in the crop row direction ranged from 0.54 to 1.04. In the direction perpendicular to the crop row, the coefficient of variation ranged from 0.06 to 1.27. The study showed insights into deriving geometric data from field crops and delivers useful information for variable equipment operations in cotton fields. Nguyen et al. [154] developed DairyBioBot, an unmanned ground vehicle (UGV) equipped with a ground-based LiDAR sensor and real-time kinematic (RTK) positioning system. In broad perennial ryegrass field experiments, this novel approach enabled accurate and effective monitoring of plant volume as a proxy for biomass. R^2^ values of 0.71 at the row level and 0.73 at the plot level showed a robust relationship between LiDAR-derived plant volume and biomass on a fresh mass (FM) basis. Robotic equipment created by Cruz-Ulloa et al. [155] made it possible to automate the fertilization process and treat each plant individually. Researchers identified cabbages as the target crop and used LiDAR technology to automate fertilizer management. Researchers reported that point clouds provide remarkable potential for exact localization, which in turn makes it possible to follow and monitor the development of specific crops over time. Machine learning methods such as Euclidean clustering [156], support vector machines, k-nearest neighbors, and k-means have often been employed for applications requiring fruit or tree measurements and phenotyping [157]. However, voxelization was used by Itakura and Hosoi [158] to recognize the weeds present in the crop.

#### 3.1.3. Crop Growth Monitoring

**Figure 14:**
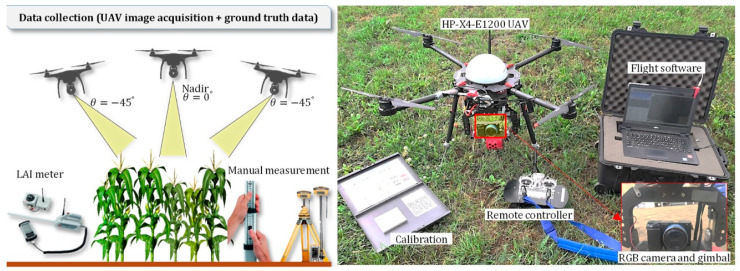
Measurement of LAI using UAV–LiDAR systems [160].

Monitoring crop growth and estimating potential yield are critical aspects of precision agriculture. LiDAR technology provides accurate and real-time data on crop height, canopy cover, and biomass. By regularly scanning fields, farmers can track the progress of crop growth and identify areas with potential issues. LiDAR data can help optimize irrigation scheduling, nutrient management, and harvest planning [159]. Many researchers recommended that the data collected through LiDAR were intended to be used by the farmer to optimize crop production, and the farmer may project the yield to get insight into the condition of the crop. Changes in crop biomass indicate to farmers that the plant has entered the reproduction phase. This shift must be noticed so that the farmer can estimate the quantity of fertilizers required in advance. Li et al. [160] found that compared to the traditional manual approach, an ALS was considerably more effective, time-saving, and useful even in difficult-to-reach locations and adverse geographic conditions for calculating LAI and plant height in maize fields. Their study emphasizes the potential of ALS technology in increasing data-gathering procedures for LAI evaluation and optimizing agricultural operations, as shown in Figure 14. With R^2^ values of 0.89, 0.86, and 0.78, the analysis showed a significant correlation between the canopy height data from the 3D point clouds and manual measurements.

#### 3.1.4. Autonomous Navigation System

For autonomous vehicles, efficient field navigation is crucial since it enables the accurate completion of various tasks. Modern autonomous vehicles use a variety of sensors to gather important information about their surroundings and current robotic state [161]. Cameras and dual-antenna real-time kinematic (RTK) systems provide a rich data stream, but their effectiveness is very sensitive to changes in lighting and environment [162]. As a result, LiDAR sensors provide improved navigational dependability in an agricultural environment, making them preferable to visual cameras [163].

Wenju et al. [164] developed an orchard-harvesting robot with autonomous navigation. The technique solved the problem of frequent navigational breaks by enabling continuous operation while turning. The method changed navigation modes based on GNSS distances. The results of the experiment were promising, with minimal data loss during communication and repeated pauses in orchard rows. The study emphasized the potential advantages of LiDAR technology for increasing navigational accuracy and orchard harvesting efficiency. To address the requirement for effective plant protection, Wang et al. [165] described the creation of an autonomous navigation system for orchards. The system used a three-module strategy: perception, decision, and control. Millimeter-wave radar and 3D LiDAR were used to identify obstacles and perceive surroundings. Based on LiDAR data, orchard navigation lines were extracted using a four-step methodology. The ADRC control method successfully improved the noise immunity of the plant protection machinery’s system. This was a great achievement in the field of agriculture technology, as it ensures that the machines can operate effectively without any interruption caused by external interference.

Liu et al. [166] combined automated navigation (AN) with precision variable-rate spraying (PVS) utilizing a single 3D LIDAR sensor to increase pesticide application safety in orchards while reducing environmental effects. Fruit trees were found, and the region of interest (ROI) was determined using a LIDAR sensor. The random sample consensus (RANSAC) technique was used in 2D processing inside the ROI to compute the center-of-mass coordinates of fruit trees and calculate the robot’s vertical distance from the fruit tree row center line. The encoder and inertial measurement unit (IMU), which correct data from the fruit tree canopy, are then used to guide the robot along the FTR center line. The findings demonstrate outstanding accuracy by drastically reducing pesticide application, air drift, and ground loss compared to conventional spraying, achieving reductions of 32.46%, 44.34%, and 58.14%, respectively. Reduced pesticide volume, ground loss, air drift, and improved environmental control result from this integrated strategy using 3D LIDAR, an encoder, and an IMU. In recent research, Bertoglio et al. [167] developed a navigation system that utilized LiDAR and wheel encoder sensors for precise navigation in row-structured agricultural environments such as vineyards. The technique used the straight, regular geometry of rows of plants. In simulated and real-world testing, the system showed mean displacement errors of 0.049 m and 0.372 m, respectively, for in-row navigation.

Additionally, the researchers devised an end-row points detection to facilitate end-row navigation in vineyards, a feature frequently neglected in comparable works. The outcomes demonstrate the efficacy and reliability of their methodology. Many algorithms, such as the graph-based optimization of simultaneous localization and mapping (SLAM) [168], RANSAC [169], and H∞ Control System [170], have also been employed for navigation-based applications. Moreover, precision agriculture has led to autonomous agricultural equipment, which decreases human labor, simplifies processes, and boosts production. However, precise obstacle detection and avoidance technologies are needed to keep these machines safe [171]. Traditional LiDAR-based obstacle identification approaches for farming areas are laborious and time-consuming owing to manually created features. To address this issue, Qin et al. [172] used deep learning on agricultural equipment with LiDAR, a camera, and GNSS/INS. Researchers used focal sparse convolution to train a 3D obstacle detector called Focal Voxel R-CNN and extracted useful features from sparse point cloud data. With a mean average precision (mAP) of 91.43% and a detection speed of 28.57 frames per second (FPS), the proposed model greatly outperformed Voxel R-CNN in terms of detection performance. This methodology presented autonomous agricultural machinery with a more dependable approach to obstacle identification. Kong et al. [173] used 2D LiDAR combined with the minimum cost maximum flow (MCMF) and density-based spatial clustering of applications with noise (DBSCAN) algorithms to identify obstacles in front of tractors. The study introduced a mechanism to classify security alert levels based on obstacle states and a way to distinguish between static and dynamic obstacles. Vehicles were tested for static and dynamic obstacles at different speeds and directions. The results revealed a remarkable average warning accuracy rate of 89.5%. The system’s reliable obstacle prediction ensures agricultural vehicle safety, advancing agricultural mechanization. Jiang and Ahmed [174] employed LiDAR to guide an autonomous spraying robot for orchard operations.

#### 3.1.5. Above-Ground Biomass (AGB) and Yield Estimation

Researchers also used LiDAR to accurately estimate above-ground biomass [175,176,177,178,179] and yield estimation [180,181]. The ability to accurately estimate canopy structure, height, and biomass distribution is useful for evaluating carbon sequestration and fine-tuning agricultural management strategies. Rapid, non-destructive data collection using LiDAR is essential for maintaining effective and sustainable agriculture methods. In southern Spain, Rodriguez et al. [182] measured carob biomass with an ALS and evaluated the crop’s carbon storage with allometric methods; this classification was essential for estimating the soil’s qualities and assessing the plants’ biomass. Sun et al. [183] used an advanced MLS to track cotton plant development throughout the seasons and year. This novel technique shed light on the dynamics of plant growth under various climatic circumstances and demonstrated how data-driven approaches have the potential to transform agricultural management practices. The successful implementation of the MLS provided thorough monitoring capabilities and has implications for improving crop yield and quality while reducing resource consumption and environmental impact, highlighting its significance in advancing productive and sustainable agricultural practices. It was also stated that yield estimates could be improved by incorporating LiDAR data.

A semi-autonomous vehicle designed for vineyard monitoring was created by Vidoni et al. [184] in response to the need for frequent estimates. Two LiDAR scanners were installed on the truck to thoroughly scan the vineyards, allowing for the study of plant volume and morphology. The researchers created a complex algorithm based on measurements of branch thickness and the NDVI. The NDVI was crucial to get important data on the properties of the tree canopy.

### 3.2. Soil Analysis and Management

LiDAR technology significantly benefits soil analysis and management practices in precision agriculture. By capturing detailed topographic information, LiDAR assists in mapping, classifying, and monitoring essential soil parameters [185].

#### 3.2.1. Soil Mapping and Classification

Combining LiDAR data with precise georeferencing techniques allows for the creation of high-resolution soil maps. By analyzing variations in surface elevation and slope from LiDAR, researchers and farmers can identify and map different soil types and their spatial distribution within a field. This detailed information supports accurate soil classification and enables targeted soil management practices, such as variable-rate fertilization and site-specific irrigation. As commercial airborne and ground-based LiDAR technology becomes more widely available, it shows tremendous potential for quantifying the spatial distribution of surface roughness (SR) across large areas reasonably and effectively [186]. By measuring the laser pulse return times transmitted from an airborne platform toward the ground, LiDAR creates a 3D point cloud. Airborne laser scanners (ALSs) can outline the geographical distribution of SR using high-density, accurate elevation data that may be quickly gathered over wide regions, providing a suitably dense sample rate [187]. Foldager et al. [188] highlighted the benefits of LiDAR technology for the efficient and precise analysis of soil surfaces in agricultural practices. Researchers measured and analyzed a furrow’s cross-sectional size and shape following a trailing shoe sweep using a manual pinboard and a LiDAR sensor. Pinboard and LiDAR measurements demonstrated significant differences of up to 41%. Additionally, LiDAR research on the effect of irrigation on furrow areas revealed variable increases in cross-sectional area under various irrigation levels. Hollaus et al. [189] used a dual approach to quantify roughness in their research and used two different measures of roughness, “Surface Roughness” (SR) and “Terrain Roughness” (TR). Full-waveform airborne laser scanning (FWF-ALS) terrain points within a certain height range from the terrain surface were measured for the SR computation. They proved that FWF-ALS helped in characterizing surface properties with its laser point attributes, and they introduced a new method, vertical roughness mapping (VRM), for identifying roughness across different vertical layers in forested regions, with accurate results when validated against in situ reference data.

#### 3.2.2. Soil Moisture Content and Nutrient Analysis

LiDAR data can indirectly provide insights into soil moisture content by analyzing vegetation reflectance patterns and canopy structure. Changes in vegetation characteristics detected by LiDAR, such as reduced canopy height or increased gaps, may indicate areas with higher moisture stress. LiDAR data, when combined with spectral analysis techniques, can also help estimate soil nutrient levels. By examining the relationship between LiDAR-derived metrics and nutrient content, farmers can develop precise fertilization plans designed to meet the demands of varying soil types throughout a field. 

Phosphorus levels are another crucial aspect of soil nutrient management, as they pose a threat to water quality. Cassidy et al. [190] employed an ALS to examine this trait in crops and mitigate the potential for excessive phosphorus in soil. The research developed a runoff routing model using LiDAR elevation data and soil hydraulic conductivity values to identify hydrologically sensitive areas. The presence of above-optimal soil phosphorus levels in agricultural fields makes places where surface runoff paths are concentrated and where the danger to water quality is greater.

To establish the appropriate resolution for defining observed soil moisture patterns, Southee et al. [191] investigated three LiDAR-derived terrain surfaces produced at different spatial resolutions. They used the topographic wetness index (TWI), percent elevation index (PEI), and canopy height model (CHM) to analyze soil moisture at spatial resolutions of 2 m, 5 m, 10 m, and 20 m, respectively. Algorithms for removing depression were also used. For depths of 0–15 cm and 0–40 cm, respectively, the coefficients of determination between soil moisture and TWI were R^2^ 0.346 and R^2^ 0.292. According to the findings, predicting soil moisture patterns at shallow depths (from 0 to 15 cm) may be more successful when using high-spatial-resolution variables (between 2 m and 5 m). On the other hand, deeper depths (from 0 to 40 cm) could be better suited for coarser resolutions (10 m, 20 m).

**Figure 15:**
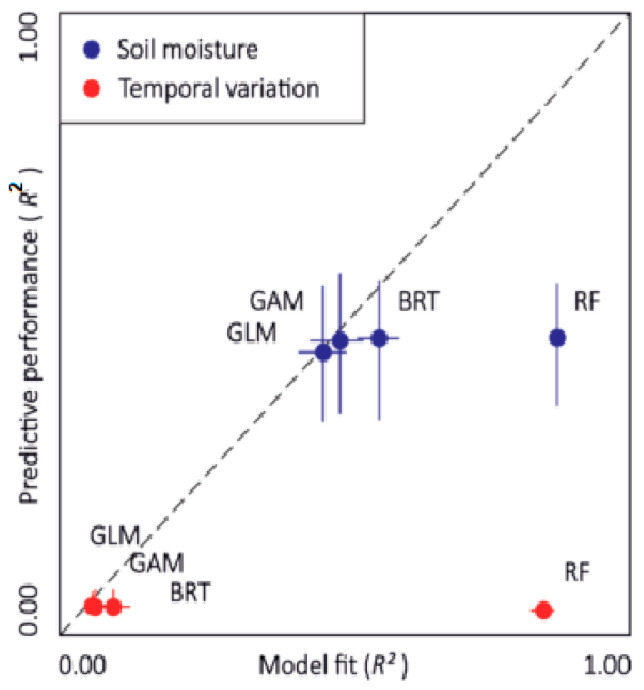
Comparing four soil moisture modeling methods [193].

To better plan water and irrigation management, Demelezi et al. [192] showed how the spatial visualization of data could show the advantages of using high-accuracy remote sensing LiDAR data to find soil hydrological characteristics and to prevent potential field waterlogging. Additionally, the utilization of remotely sensed data, such as LiDAR, provided a significant benefit in data collection and site information presentation. As a result, at the plot-farm level, mapping and digitalizing landscape LiDAR information was beneficial, accurate, and simple to comprehend. Comparatively, Kemppinen et al. [193] used LiDAR data and field experiments to examine the role of soil and land surface variables on landscape-scale soil moisture change. This investigation modeled soil moisture and its temporal change using generalized additive models (GAM), boosted regression trees (BRT), and RFR. Figure 15 presents the forecasting abilities of four different soil moisture modeling techniques. The horizontal and vertical segments show the ranges of each modeling technique. Results showed that the average predictive performance was an R^2^ value of 0.47 and an RMSE of 9.34 VWC (volumetric water content, %), whereas the average model fit was an R^2^ value of 0.60 and an RMSE of 8.04 VWC%. The temporal variation models performed predictably with an R^2^ of 0.01 and an RMSE of 15.29 CV%, and they fit with an R^2^ of 0.25 and an RMSE of 13.11 CV%.

## 4. LiDAR Applications in Crop Harvesting

Real-time crop production process optimization, including fertilization, crop protection, and harvesting, becomes possible when these essential aspects are considered. However, research and development on the vehicle-based use of LiDAR technology in agriculture for collecting and exploiting such characteristics is still in the early stages. This section explores how LiDAR technology is utilized in various aspects of crop harvesting to improve efficiency and productivity. We discuss three key areas of application: crop maturity and yield prediction, autonomous harvesting systems, and post-harvest quality assessment.

**Figure 16:**
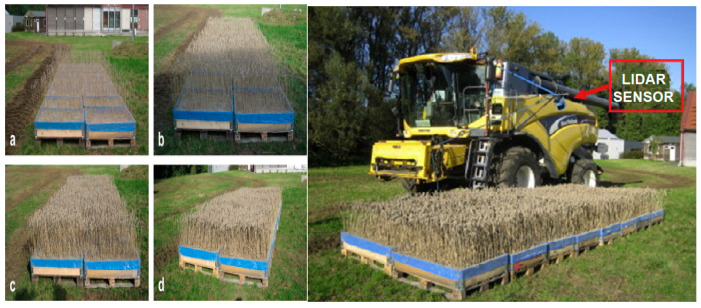
Different crop densities and combine harvester with LiDAR system (**a**) 100 ears per m^2^; (**b**) 200 ears per m^2^; (**c**) 300 ears per m^2^; (**d**) 400 ears per m^2^ [194].

Saeys et al. [194] investigated how to assess crop density in front of combine harvesters using two LiDAR sensors and data-processing techniques. An illustration of the crop density and combine harvester mounted with the LiDAR system is depicted in Figure 16. It was shown that using a linear model based on the local standard deviation of the penetration depth, accurate estimations of crop density could be generated from the scans from both sensors. R^2^ values for this linear model varied from 0.81 to 0.96 during experiments with various driving speeds and machine vibration levels. The suggested new approach for predicting crop volume showed excellent results. Researchers concluded LiDAR technology could improve combine harvester automated feed rate control systems.

Selbeck et al. [195] investigated the vehicle-based scanning LiDAR sensor in terms of its measuring properties in maize stands. Laser beam form, laser layer, laser echoes, field of view, and data density variation were all mentioned as potential sources of measurement error, and solutions were proposed to eliminate them. The 3D model made from the scanner data allowed the computation of the volume and biomass parameters for machine control and gave adequate surface information about maize stands. The researchers concluded that there is a correlation between the weighted biomass and the height and volume calculated by the model. Deremetz et al. [196] suggested an ultra-wide-band-based technique to guarantee a relative localization without requiring a direct line-of-sight to the object. To replicate a person’s route without direct communication or an absolute localization system, the approach locally approximates the trajectory of the human leader as a circle. Prins and Niekerk [197] used LiDAR, Sentinel-2, aerial, and machine learning to distinguish five crop varieties in an intensively planted region. Ten machine-learning techniques and various combinations of the three datasets were tested. The classification results were interpreted by comparing the aggregate accuracies, kappa, standard deviation, and f-score. Using LiDAR data to distinguish between various crop types was effective, with XG Boost offering the most significant overall accuracy of 87.8%. Furthermore, the crop-type maps made using Sentinel-2 data and those created using LiDAR data largely agreed.

To determine the optimal time to harvest sugarcane, Canata et al. [198] investigated LiDAR technology, specifically a laser sensor mounted on agricultural platforms. The measurement system integrates a laser sensor, GNSS receiver, and inertial device with a computer on the platform of an agricultural tractor. The data collection process was carried out around ten days before harvest. The approach generated point clouds with a density of roughly 2000 points m^−2^ and extracted sugarcane plant height measurements. The research discovered that platform oscillation vibration considerably impacted the dataset in one experimental area owing to high-amplitude spectral power. The suggested measuring system identified sugarcane plants in the pre-harvest phase without signal saturation issues.

### 4.1. Crop Maturity and Yield Prediction

**Figure 17:**
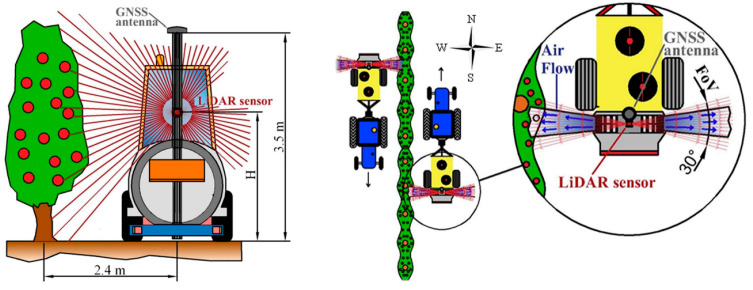
Multi-beam LiDAR system for the detection of fruits [200].

LiDAR technology offers non-destructive and accurate methods for crop maturity and yield prediction. By capturing detailed 3D data of crop canopies, LiDAR enables precise assessment of key parameters such as plant height, leaf area, and canopy density. These metrics are critical for determining crop maturity and optimizing harvest timing, thereby maximizing crop quality and yield potential. LiDAR-based yield estimation models have shown promising results in predicting crop yields with high accuracy, contributing to better planning and resource allocation. To forecast wheat production and grain protein content (GPC), Sun et al. [199] used LiDAR technology with deep learning algorithms and time-series proximate sensing data. They incorporated LiDAR data using data fusion methods, which significantly increased prediction accuracy. Apple fruit detection and yield prediction were accomplished by Gene-Mola et al. [200] using a multi-beam LiDAR sensor with forced airflow and an air-assisted sprayer. The fruit identification algorithm was developed using reflectance thresholding (RT) and a support vector machine (SVM). The experiment was designed to boost fruit detection and decrease fruit occlusions by shifting the tree foliage using a multi-view sensor. Figure 17 illustrates how forced airflow affects fruit identification.

When comparing the number of apples found by LiDAR with the forced airflow system to the real number of apples per tree, the RMSE was 19.0%, and the R^2^ was 0.58 and 0.54, respectively, when scanning was performed from the east and west sides. The RMSE was 5.7%, and the R^2^ was 0.87 when both sides of the trees were evaluated. As a surrogate for biomass, canopy height has been widely adopted [201]. Tilly et al. [202] added a field spectrometer to their platform and derived bivariate biomass regression models by fusing 3D data and spectral TLS data. Researchers used linear and exponential biomass regression models (BRMs) to evaluate the accuracy of plant height and vegetation indices (VIs) as fresh and dry biomass estimators. Their results demonstrate the promising potential of remotely sensed plant characteristics for estimating barley biomass. Li et al. [203] investigated the potential of airborne-LiDAR data in measuring the AGB and belowground biomass (BGB) of maize; it identifies canopy height and LAI as essential factors that directly affect biomass components. LiDAR-derived maize height and LAI data were used to illustrate the geographical distribution of biomass components and field-based estimate methods were presented. The findings showed that LiDAR-estimated biomass was equivalent to field-measured biomass, highlighting the enormous potential of airborne LiDAR for determining canopy height, LAI, and the various biomass components of maize at the height of the growth season.

### 4.2. Autonomous Harvesting Systems

**Figure 18:**
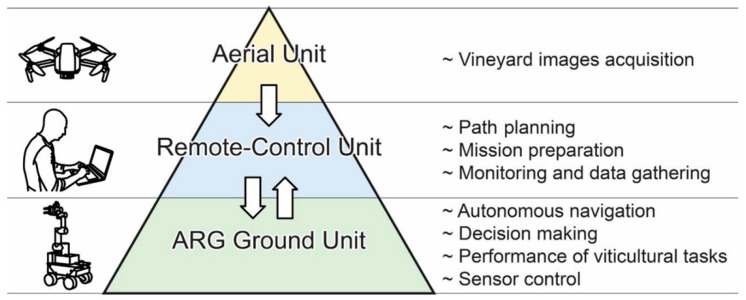
Conceptual architecture system of the integration of three parts [207].

LiDAR technology is essential for the advancement of autonomous harvesting systems. It provides real-time, precise data on crop location, size, and orientation, enabling autonomous vehicles and robotic systems to navigate and maneuver with high accuracy in agricultural fields. By employing both 2D and 3D LiDAR, these systems can effectively detect and avoid obstacles, including plants, structures, and other potential hazards, minimizing the risk of collisions. This capability enhances the safety and efficiency of harvesting operations, allowing for continuous, high-quality crop collection while reducing the need for manual intervention. Furthermore, the integration of LiDAR data with other sensors and advanced algorithms improves decision-making processes, optimizing the overall performance and productivity of autonomous harvesting systems [204,205]. Additionally, LiDAR-based perception systems enhance the precision and dexterity of harvesting robots, enabling them to harvest ripe crops selectively while minimizing damage to the surrounding vegetation. Geer et al. [206] developed a LiDAR-based SLAM autonomous robotic system for harvesting strawberries with a novel software architecture, GMapping version 1.3, integrated with ROS and a SLAM algorithm. Laboratory and field experiments were conducted to assess the accuracy of the autonomous harvesting system. Researchers reported that advanced robotic systems could identify the correct rows to travel between while maintaining a constant distance from the fruit trees. The perception system was designed to function effectively, avoiding fruit damage or loss of position. Vrochidou et al. [207] developed a LiDAR-based autonomous harvester for grape harvesting, which mainly consisted of three units: (1) an aerial unit, (2) a remote-control unit, and (3) the ARG ground unit, as shown in Figure 18. Three machine vision algorithms-grape cluster detection, ripeness assessment, and grape stem identification—were sequentially executed to complete the harvesting process. LiDAR utilized two algorithms for localization: (1) an ICP algorithm for mapping robot pose utilizing 3D data from 16 laser beams to ensure precise position monitoring, and (2) a wall-following algorithm using one LiDAR laser beam to maintain a fixed operational distance. Researchers have also confirmed that the inherent versatility built into the system’s architecture enables it to be seamlessly adapted for various crops and orchard settings.

Xiong et al. [208] designed a fully integrated LiDAR-based strawberry harvesting system for picking strawberries in clusters. The data from the 2D LiDAR were processed using the GMapping SLAM technique to generate a map. The system was tested on a strawberry farm, demonstrating its capacity to effectively harvest strawberries that were partly covered by leaves. Depending on the parameters, the first-attempt success rates ranged from 50.0% to 97.1%, while on the second try, these rates rose to 75.0–100.0%.

### 4.3. Post-Harvest Quality Assessment

After harvesting, maintaining the quality of crops is crucial for maximizing their market value. LiDAR technology offers valuable insights for post-harvest quality assessment. By analyzing the optical properties of harvested crops, such as color and surface characteristics, LiDAR can help detect defects, damage, or signs of spoilage. This information enables the timely sorting and grading of crops, ensuring that only high-quality products reach the market. Furthermore, LiDAR-based 3D scanning can facilitate precise crop volume and shape measurements, providing essential data for efficient packaging and storage. Many researchers used LiDAR technology to quantify crop height and wheat straw production for post-harvest quality evaluation [209,210]. When utilized along with yield monitor data, this might be very helpful in calculating and mapping signs of crop stress for the post-harvest evaluation of growing season conditions.

**Figure 19:**
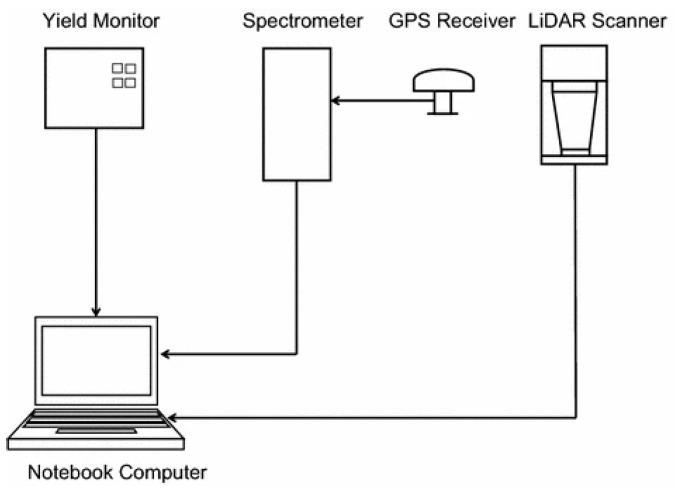
Multi-sensor system for post-harvest assessment of environmental stress in wheat [208].

Long et al. [211] used a hybrid strategy, combining a multi-sensor system with LiDAR sensor technology to present accurate, site-specific measures of grain production, grain protein content, and straw yield. The system included a mass-flow yield monitor, an optical NIR spectrometer, a LiDAR sensor, a global positioning system receiver, and a laptop computer, as shown in Figure 19. This combined method maintained the same level of spatial resolution as grain yield. By analyzing connections between grain yield and protein maps produced by the yield monitor and protein sensor, researchers could detect areas in fields where nitrogen or water stress was limiting grain output. The results of this study highlight the potential of stress-related indicator maps in enhancing the ability to evaluate grain yield maps.

Mulley et al. [212] investigated the potential for determining the health of date palms using accessible thermal, hyperspectral, LiDAR, and visual RGB pictures. Despite difficulties in preprocessing and evaluating the quality of hyperspectral and thermal data, the approaches used in this inquiry provide interesting directions for future investigations. These results highlight how remote sensing data may support precision agricultural techniques and improve plantation management. Notably, the combined use of high-resolution thermal and hyperspectral images may provide information on the health of specific trees. Additionally, combining these indicators may provide a complete tree health assessment technique. Table 5 summarizes different LiDAR systems and LiDAR sensors used for efficient crop management by different researchers.

From the summary provided in Table 5 above, it is observed that, due to their easy access to crops, MLSs are useful for a variety of tasks, including crop monitoring, object detection and classification, tree volume estimation, crop monitoring, and navigation. On the other hand, the TLS has a distinct advantage in tasks such as pruning and is particularly suitable for digitization-related tasks such as accurately capturing tree structure and foliage. For both the MLS and TLS, the following LiDAR sensor types are often used: LMS511, LMS111, Focus 350, LMS511, and VLP-16 (also known as Puck). Regarding duties such as tree counting, determining irrigation areas, assisting navigators, and monitoring orchards, ALSs excel because of their unique viewpoint and are excellent at capturing the spatial arrangement of things. When it comes to processing point clouds, CloudCompare, LiDAR360, and Point Cloud Library are the three most used tools, and the most prevalent sensor type is the VLP-16. ArcGIS is a frequently used option for viewing and analyzing LiDAR point cloud rasterization data. By utilizing point cloud processing techniques, one can estimate metrics, classify and cluster data for artificial vision and monitoring purposes, and voxelize digital representations of foliage, seeds, plants, and tree structures. This enables an integrated approach to tasks utilizing LiDAR technology. Table 6 provides a comprehensive comparison between different types of LiDAR systems (ALS, TLS, and MLS) and their respective advantages, applications, and limitations for crop management in precision agriculture.

## 5. Conclusions

In conclusion, this review paper explored the critical evaluation of LiDAR applications in precision agriculture for crop management. LiDAR technology has shown great potential in revolutionizing crop management practices in precision agriculture. Its applications in crop yield forecasting, biomass mapping, crop height measurement, and autonomous navigation for agricultural robots demonstrate its ability to provide accurate and timely data for decision-making. As LiDAR technology advances, it is expected to play an increasingly important role in optimizing cultivation and harvesting practices, ultimately contributing to more sustainable and efficient agricultural systems. LiDAR technology emerged as a promising tool with its unique capabilities and advantages in agriculture. This section explores the future perspectives and challenges associated with LiDAR applications in precision agriculture and the potential advancements and innovations that will likely shape the field.

### 5.1. Emerging Trends and Innovations in LiDAR Technology

This subsection focuses on the emerging trends and innovations in LiDAR technology that are relevant to precision agriculture. New developments in LiDAR technology for precision agriculture encompass a range of advancements. These include downsizing and integrating LiDAR sensors onto drones and equipment, resulting in enhanced compactness and durability. Moreover, higher resolution and multi-wavelength capabilities significantly improve object recognition. Sensor fusion has enhanced environmental modeling accuracy, while integration with autonomous vehicles and advanced driver-assistance systems has opened new avenues for real-time crop monitoring in precision agriculture. Combining LiDAR with data from other sensors allows for comprehensive crop health and environmental assessments. Additionally, LiDAR, improved by machine learning and AI, facilitates automated crop management, variable rate application, and 3D modeling. Additionally, autonomous vehicles and robots with LiDAR are transforming processes such as planting and harvesting, and cloud-based data processing and accessibility make precision agriculture accessible and cooperative. These emerging trends highlight the evolving nature of LiDAR technology and its potential to revolutionize precision agriculture.

### 5.2. Key Challenges and Research Gaps

In this subsection, we address the key challenges and research gaps that exist in the implementation of LiDAR technology for precision agriculture. We discuss obstacles such as data processing and interpretation, sensor calibration and accuracy, and cost-effectiveness. Additionally, we explore the need for further research in data fusion techniques, standardized protocols, and long-term impact assessments.

Despite the promising applications of LiDAR in precision agriculture, several challenges remain. The main challenge with using LiDAR technology in agricultural applications is the initial capital cost, which is often significant. To address this, there is a need for cost-effective LiDAR sensors designed specifically for research applications that offer a balance between affordability and performance. Researchers must also devise methods to fully leverage the capabilities of these more economical sensors to facilitate broader adoption in the agricultural sector. In addition, the operational and processing difficulties associated with LiDAR data and the limited canopy penetration capacity for low-stature vegetation such as maize also present a research gap in LiDAR application in agriculture.

### 5.3. Potential Solutions and Future Directions

To overcome existing challenges, further research and development are needed to improve LiDAR technology and its integration with other remote sensing techniques. Furthermore, the development of automated, on-the-go data processing capabilities and specialized commercial LiDAR systems for field operation may aid the use of LiDAR in precision agriculture. One of the key areas of research is to improve data processing efficiency, which is crucial for handling large quantities of data collected by LiDAR sensors. Researchers are now studying expert algorithms and computational approaches to streamline data processing and analysis to facilitate the rapid and accurate estimation of biomass components. Recent research also covers sensor calibration techniques, which are essential to ensure the accuracy and reliability of LiDAR data. Calibration methods are being improved to decrease data collection errors and increase biomass estimation accuracy.

Cost saving is another important consideration when using LiDAR technology for crop parameter estimates. Researchers are investigating novel approaches to reduce costs and improve the affordability of LiDAR systems for farmers and agricultural practitioners. Reducing costs while increasing data quality involves analyzing alternate sensor designs, using drone-based LiDAR platforms, and optimizing data gathering techniques.

We stress the value of multidisciplinary interactions in addition to technological developments. The issues faced by LiDAR-based crop management techniques might be addressed comprehensively by bringing together professionals from multiple disciplines, such as remote sensing, agronomy, and data science. Researchers may design integrated solutions that use the full capabilities of LiDAR technology in agriculture by combining their skills, knowledge, and resources. LiDAR technology can potentially improve the performance of other precision agricultural tools. LiDAR data may be used with data from drones, remote sensing satellites, and ground-based sensors to provide a thorough and precise picture of crop management practices. This connection enables rapid and well-informed decision-making.

## Figures and Tables

**Table 1:** Uses of ALSs in the realm of crop management.

Crop	Crop Management Practices	Important Findings	Ref.
Sorghum	Growth monitoring	UAV–LiDAR and RGB photogrammetry accurately assessed canopy height (CH) and LAI in sorghum. LiDAR outperformed photogrammetry, achieving superior accuracy with an R^2^ of 0.975 and a RMSE of 5.94% for CH.	[71]
Pea, Chickpea	Crop height estimation	Highly significant correlations (*p* < 0.0001) were found between LiDAR-estimated and manually measured plant heights, with correlation coefficients (r) of 0.74 for chickpeas and 0.91 for peas.	[72]
Atlas, Red Carina, Pasto	Height, biomass, and grain estimation	The LiDAR-based hyperspectral physiological reflectance index (PRI) demonstrated superior performance, distinguishing between salt-affected and treated plants. The overall dataset achieved an R^2^ of 0.46, with specific subgroups reaching an R^2^ of 0.64.	[73]
Potato	Monitoring agricultural biomass and plant growth	The LiDAR scanner demonstrated a strong correlation with field-measured parameters, with a high R^2^ of 0.89 and a low RMSE of 0.028 m for PH, and an R^2^ of 0.81 with an RMSE of 31.65% for AGB.	[74]
Sugarcane	Prediction of biomass and leaf N_2_ content	Crop growth parameters were monitored, including height, density, and vegetation indices. Predictive models were evaluated based on multispectral predictors alone, LiDAR predictors alone, and a fusion of both, then compared against an NDVI benchmark. The multispectral model showed slightly superior performance (R^2^ = 0.57) compared to the LiDAR model (R^2^ = 0.52), with both models surpassing the NDVI benchmark (R^2^ = 0.34).	[75]
Sugarcane	Estimation of yield and aboveground fresh weight	Six regression algorithms (MLR, SMR, GLM, GBM, KRLS, and RFR) were employed to construct the sugarcane aboveground fresh weight (AFW) model using LiDAR data. Results showed that RFR outperformed other models’ prediction accuracy (R^2^ = 0.96, RMSE = 1.27 kg m^−2^). The final sugarcane AFW distribution maps demonstrated strong agreement with detected values (R^2^ = 0.97, RMSE = 1.33 kg m^−2^).	[76]
Sorghum	Biomass estimation and prediction	Two biomass prediction methods were explored: one utilized LSM to predict biomass from LiDAR point cloud data directly, and the other employed the APSIM crop simulation model. The LiDAR approach yielded R^2^ values of 0.48 and 0.55 for SVR and MLP, respectively, while the APSIM model achieved R^2^ of 0.31 and 0.67 for SVR and MLP, respectively.	[77]
Avocado, Macadamia, Mango	Orchard and tree crown assessment	The study found that crown structure measurements, particularly those based on the top of the crown, exhibited strong consistency between ALS and TLS data. Crown area measurements showed the highest correlation (R^2^ = 0.997) between the two data sources. The linear model’s RMSE for maximum crown height derived from ALS and TLS data was 0.29 m, with an R^2^ of 0.99.	[78]
Wheat	Monitoring of CH, biomass, and N2 uptake	The 95th percentile of normalized LiDAR points showed a strong correlation (R^2^ = 0.88) with manually measured crop heights and (R^2^ = 0.92) with crop heights obtained from a UAV system using optical imaging, out of the 57 UAV–LiDAR metrics that were analyzed.	[79]
Sorghum	Biomass prediction	Geometric features extracted from LiDAR data yielded dependable and precise biomass predictions. The 750–1100 nm spectral range proved to be the most informative for biomass prediction, with R^2^ values for end-of-season biomass ranging from 0.64 to 0.89.	[80]

**Table 2:** Classification, implementation, and limitations of airborne LiDAR systems.

Component	Description	Implementation	Limitations
LiDAR Sensors	Emits laser pulses and measures the time it takes for them to return	Mounted on aircraft (e.g., UAVs and planes)	Limited by battery life and flight duration (especially for UAVs)
Global Positioning System (GPS)	Provides precise location information	Integrated with LiDAR sensor	Signal interference can affect accuracy, especially in dense vegetation
Inertial Measurement Unit (IMU)	Measures the rate of acceleration and changes in rotational attributes	Works with GPS to provide accurate positioning data	Sensor drift over time can affect data accuracy
Data Storage	Onboard storage system for capturing LiDAR data	High-capacity storage systems onboard	Storage capacity may limit the amount of data collected during a single flight.
UAV (Unmanned Aerial Vehicle)	Aircraft without a human pilot onboard are used for carrying LiDAR sensors	Suitable for small to medium-scale areas	Limited flight time and payload capacity; subject to weather conditions
Manned Aircraft	Airplanes or helicopters piloted by humans are used for carrying LiDAR sensors.	Suitable for large-scale mapping	Higher operational costs and regulatory restrictions

**Table 3:** Use of TLSs in the realm of crop management.

Crop	Crop Management Practices	Important Findings	Ref.
Barley	Plant-height measurement	A comparison was made between TLS and UAV-based imaging for CSM-derived plant height in crops using analyses based on polygon grids. The results revealed a high correlation between TLS and UAV-derived plant height (R^2^ = 0.91), with a 4.81% higher coefficient of variance observed in TLS compared to UAV data.	[89]
Wheat	High-throughput phenotyping of plant height	The comparison of PH derived from LiDAR and structure from motion revealed high consistency, with a strong correlation (R^2^ ≈ 0.98) and minimal RMSE values (RMSE = 8.4 cm). LiDAR and structure from motion exhibited high repeatability (H^2^) in plant height.	[90]
Rice	Biomass estimation for individual organs and aboveground biomass (AGW)	Three regression approaches, SMLR, RF, and LME modeling, were assessed for biomass estimation using an extensive TLS. The LME model exhibited the most significant improvement in panicle biomass, showing a 0.74 increase in R^2^ (LME: R^2^ = 0.90, SMLR: R^2^ = 0.16) and a 1.15 t/ha decrease in RMSE (LME: RMSE = 0.79 t/ha, SMLR: RMSE = 2.94 t/ha). In comparison to SMLR and RF, LME modeling provided similar AGB estimation accuracies for pre-heading stages but notably higher accuracies for post-heading stages (LME: R^2^ = 0.63, RMSE = 2.27 t/ha; SMLR: R^2^ = 0.42, RMSE = 2.42 t/ha; RF: R^2^ = 0.57, RMSE = 2.80 t/ha).	[91]
Wheat	Estimation of plant height, ground cover, and AGW	Canopy height strongly correlated with LiDAR (R^2^ = 0.99, RMSE = 0.017 m). In contrast to NDVI, LiDAR remained unaffected by saturation at high ground cover and exhibited a strong association (R^2^ = 0.92, slope = 1.02) at ground cover above 0.8. AGW estimation employed 3D voxel index (3DVI) and 3D profile index (3DPI), with the strongest associations with biomass observed for 3DPI (R^2^ = 0.93) and 3DVI (R^2^ = 0.92).	[92]
Vineyard	Canopy characterization (crop height, width, volume, and leaf area)	Ultrasonic and LiDAR sensors were compared to manual canopy measurements. Strong correlations were found between crop volume values from ultrasonic sensors and leaf area index (R^2^ = 0.51) and canopy volumes measured by ultrasonic and LiDAR sensors (R^2^ = 0.52). LiDAR accurately predicted canopy volume.	[93]
Grapevine	Canopy geometry characterization	Significant correlations emerged between LIDAR impacts and LAI during each growth stage. The estimated values of tree row volume (R^2^ = 0.99) and leaf wall area (R^2^ = 0.95) exhibited statistical significance relative to the vine’s growth stage.	[94]
Apple, Pear, Vineyards	Measurement of non-destructive vegetative volume and surface area of tree row	Strong correlations were found between manual and LIDAR-based measurements of vegetative volume in tree-row plantations, especially with the tree area index (TAI) parameter. A significant correlation (R^2^ = 0.814) was also observed between LIDAR volume and foliar area.	[95]
Maize	Detection and discrimination of plants and weeds	A high correlation (R^2^ = 0.75) existed between LiDAR-measured height and actual plant height. Achieving up to 95% accuracy, the LiDAR-based sensor effectively discriminated vegetation from the ground. Weed discrimination success was demonstrated through canonical discriminant analysis (CDA) with a rate of 72.2%.	[96]
Vineyard, Apple	Monitoring of pesticide clouds	The LiDAR system recorded the mid-range spray drift with a 2.4 m distance resolution and a 100 ms temporal resolution at the highest pulse repetition frequency.	[97]
Vineyard	Drift detection in vineyard spraying	At an airflow rate of 34,959 m^3^/h, correlation coefficients for conventional nozzles ranged from 0.87 to 0.91, while for air injection nozzles, the range was from 0.88 to 0.40. At 27,507 m^3^/h, conventional nozzles showed coefficients between 0.85 and 0.94, whereas air injection nozzles ranged from 0.07 to 0.88. Finally, at 6423 m^3^/h, conventional nozzles had coefficients from 0.93 to 0.98.	[98]

**Table 4:** MLS application in crop management.

Crop	Crop Management Practices	Important Findings	Ref.
Apple	Estimation of tree canopy density	The 2D algorithm identified peak points at the canopy center, correlating with apple variety, tree age, and tall spindle form. Notably, the middle section exhibited higher canopy points than the top and bottom. The 3D algorithm excelled in evaluating tree canopy point density, surpassing the 2D version. Ensuring precise alignment during scanning is vital to avoid experimental errors.	[109]
Wheat	Crop biomass estimation	A proximal active reflectance sensor offering spectral indices and crop height estimates was compared to the LiDAR system. The correlation between LiDAR-derived crop height and crop biomass was 0.79, revealing substantial variability in biomass across the field. This suggested the potential of LiDAR technology for large-scale operations and site-specific management.	[110]
Apple	Leaf area detection	A test platform was constructed to measure orchard tree canopy leaf areas manually. Polynomial regression, BPNN, and PLSR algorithms were employed to analyze the relationship between canopy point clouds and leaf areas. The BP neural network (86.1% test, 73.6% verification accuracy) and PLSR (78.46% test, 60.3% verification accuracy) outperformed the Fourier function in polynomial regression (59.73% accuracy).	[111]
Vineyard	Estimation of canopy size parameters (thickness, height, and volume) and LAI	UAVs, MLSs, and mobile apps (MA) effectively estimated canopy size variations. Strong correlations (R^2^ > 0.7) were observed, with the highest at R^2^ = 0.78 (UAV vs. MLS) for canopy volumes. Height data showed robust correlations (R^2^ = 0.86, MA vs. MLS), while thickness data had weaker correlations (R^2^ = 0.48, UAV vs. MLS). LAI demonstrated moderate but consistent correlations with canopy volumes, ranging from R^2^ = 0.69 (LAI vs. UAV) to R^2^ = 0.74 (LAI vs. V MLS).	[112]
Sorghum	Detection and measurement of estimation of individual sorghum panicles	Panicles were identified with 89.3% overall accuracy, encompassing a 10.7% omission and 14.3% commission rate. Estimated panicle dimensions demonstrated a strong correlation with LiDAR-derived measurements (panicle length: r = 0.88, RMSE = 3.10 cm; panicle width: r = 0.79, RMSE = 1.67 cm; plant height: r = 1.00, RMSE = 0.80 cm). Comparison with harvested panicle data revealed moderate-to-high correlations (panicle length: r = 0.79, RMSE = 2.48 cm; panicle width: r = 0.63, RMSE = 1.49 cm; plant height: r = 0.86, RMSE = 11.4 cm).	[113]
Cabbage, Leek, Potato, Wheat	Canopy estimation	Soil and plant segregation was accomplished by calculating weighted sums, eliminating the need for additional sensor data. This dynamic method extracted vegetation from point clouds in strips with varying coverage and sizes. The resulting vegetation clouds were validated against drone imagery, confirming a precise match with all green areas.	[114]
Ryegrass	Estimation of fresh weight and dry matter yield	R^2^ between FWY and seasons (winter, spring, summer, and autumn) were 0.81, 0.92, 0.94, and 0.90, respectively. Similarly, the R^2^ values between DMY and the seasons were 0.87, 0.73, 0.87, and 0.79, respectively. These results suggest that LIDAR estimation of DMY is accurate within seasons for paired-row breeding plots. However, it is sensitive to significant changes in dry matter content (%) among seasons, requiring seasonal algorithms for correction.	[115]
Miscanthus giganteus	Measurement of crop height	The sensor assessed stem densities in static mode, yielding an average error of 5.08% (max 8%, min 1.8%). It also measured crop height in a 5 × 10 m field, showing a 4.2% error compared to manual measurements. The sensor traversed a field edge in dynamic mode, generating a three-dimensional crop structure. An ordinary least-squares surface-fitting algorithm produced top and ground surfaces, resulting in an average crop height. Dynamic measurements showed a 3.8% average error (max 6.5%, min 1.5%).	[116]

**Table 5:** Summary of the use of LiDAR systems for efficient crop management.

LiDAR Types	LIDAR Sensor	Software System	Algorithm	Model	Crop	Feature	Ref.
TLS	RIEGL VZ-1000	RiSCAN Pro 8.0	ICPA IDW	MCSM, DEM	Paddy rice	Crop height	[213]
TLS	ILRIS	TerraScan 16.0 Surfer 8	ICP	QTM	Shrubs	Biomass detection	[214]
TLS	RIEGL VZ-1000	RiSCAN Pro 8.0	ICP	CSM, DTM, CHM	Wheat	Canopy hight detection	[215]
ALS	VUX-1UAV	TerraScan 16.0 R Studio	Random forest	CHM, DEM, DSM	Maize, Soybean	Crop heights estimation	[216]
ALS	Leica ALS70	TerraScan 16.0 ENVI 5.6	TIN FLASH	DTM, BEM	Maize	Biomass estimation	[217]
ALS	DJI Matrice M300	CloudCompare 2.11.3 DJI Terra 3.0 Python 3.7 LiDAR 360	TransUNet U-Net, FCN PCTA CXHA	CMM, DSM, DTM	Broccoli	Canopy and head estimation	[218]
TLS	SICK LMS200	CloudCompare 2.11.3 MATLAB R2013a R Studio 0.96	-	DTM	Sugarcane	Monitoring production	[219]
TLS	RigelScan Elite	PCL 1.9.1	RANSAC PCPA CGA	-	Legume	Phenotyping	[220]
ALS	RIEGL RiCOPTER	CloudCompare 2.10.2 RiPROCESS 4.0, RiPRECISION 4.0	3DPI	DTM, DSM, CHM	Potato, Sugar beet, Wheat	Crop height and biomass estimation	[221]
TLS	LMS 511, SICK AG	LabVIEW 2.0 MATLAB 2016b	PCPA K-means clustering LOWESS	3DLSM	Sorghum, Maize	Morphological traits detection	[222]
TLS	FARO Focus S70	SCENE 2022 PCL 1.12.0	DBSCAN RNN KDTree	MSM MOSM	Maize	Segmentation and stratification	[223]
TLS	LMS-Z420i	RiSCAN Pro	SDA	CSM, DTM, DSM,	Barley, Sugar beet	Crop heights estimation	[224]
ALS	RIEGL VUX-1UAV	LiDAR360	-	CHM, DEM, DTM, DSM	Maize	Lodged crop height estimation	[225]
TLS	Kaarta Stencil 2	CloudCompare 2.12.4 MATLAB 2022a	CXHA ASA	-	Olive	Tree volume estimation	[226]
TLS	RIEGL LMS-Q680i	SideLook 1.1.01 R Studio 1.0	Mean height	DTM, CMM	Corn	Hail defoliation assessment	[227]
TLS	FX6 LIDAR	PCL 1.0 MATLAB R2011a	RANSAC	CRM	Maize	Plant detection and mapping	[228]
TLS	LiDAR Lite V3	R Studio PCL Arduino IDE 1.8.13	-	IWM	Palm	Bunch ripeness prediction	[229]
TLS	FARO Focus 3D X120	FARO SCENE 5.4.4 LiDAR360	-	DTM,	Maize	Monitoring phenotypes	[230]
TLS	Velodyne VLP-16	VeloView 3.5 MATLAB	RANSAC	LBM	-	Plowing furrow recognition	[231]
TLS	LiDAR-Lite v3 LMS400	CloudCompare Python 2.7 MATLAB 2017a	PCSA PCAA	3D point cloud	Canola	Leaf morphology extraction	[232]
TLS	TDS-130L 3D	Point Cloud MeshLab	RANSAC ICPA	-	Tomato	Leaf morphology analysis	[233]
TLS ALS	FARO Focus 350 RIEGL VUX-1UAV	CloudCompare2.11.3 RiProcess 2.8 FARO SCENE POSPAC UAV 5.0	-	LADE	Maize	Ear height estimation	[234]
ALS	Leica Aibot X6V2	CloudCompare 2.9.1 MATLAB R2019a	ASA PCAA	DTM, DEM	Apple	Crown Parameters detection	[235]
ALS	Leica ALS50-II	FUSION/LDV 3.42	Tree detection algorithm	DTM, CHM	Olive	Pruning biomass estimation	[236]
ALS	LM7800	CloudCompare, MATLAB, FUSION/LDV	ASA, k-means algorithms	DTM, DSM	Walnut	Crown parameters detection	[237]
MLS	LMS511 pro	CloudCompare 2.10.2 MATLAB R2021a	ICPA CHA	-	Strawberry	Plant vegetative growth	[238]
TLS	3D Velodyne, 2D Sick LMS	PCL 1.11.1 CloudCompare 2.10.2 Pix4D 4.6.0	ICPA RANSAC	KM, DEM	Citrus	Canopy height	[239]
TLS	LMS-511	CloudCompare 2.11.0 MATLAB R2022a	ICPA	3D Point Cloud LRM, KNN	Apple	Tree canopy and fruit quality	[240]
TLS	SICK LMS111	LabVIEW 2020 MATLAB R2020a	ICPA	CSM, CHM, DSM	Apple	Canopy leaf area	[241]
TLS	Sick LMS-111	MATLAB R2022a	ASA	-	Peach	Tree canopy estimation	[242]
TLS	Sick LMS200	PCL 1.8.1 MATLAB R2018a	LRM	-	Apple, Pear, Vineyard	Canopy leaf area	[243]
TLS	OMD10M-R2000-B23-V1V1D	Python 3.7 LabVIEW 2019	SORA GNBA	URL, PLSRM	Banana	Fruit chlorophyll estimation	[244]
ALS	Velodyne Puck LITE	PCL CloudCompare 2.11.3 MATLAB R2021a	ICPA SLAM	CHM, LAI	Peach	Plant parameter estimation	[245]
TLS	Sick LMS 200	CloudCompare 2.8.1 R Studio 1.1.x	ASA CXHA	-	Orange	Canopy volume estimation	[246]
TLS	Sick AG LMS111	SOPAS ET MATLAB R2016a	PCA	-	Plants	Plant monitoring	[247]
TLS	Optech ILRIS 36D	Polyworks v10	-	LLSVM	Grapevine	Biomass estimation	[248]
MLS, TLS	Backpack DG50 FARO Focuss 350	CloudCompare 2.6.1 MATLAB 2016a LiDAR 360 v4.1	TreeQSM	QSM	Apple	Tree branch information	[249]
MLS	VLP-16 LiDAR	CloudCompare 2.9.1 MATLAB 2016a	CXHA, CSF ASA VBS ASBS	DTM	Grapefruit	Canopy parameter estimation	[250]
TLS	FARO Focus 3D	CloudCompare MATLAB TreeQSM FARO SCENE v2022.1.0	-	QSM	Peach	3D crown architecture estimation	[251]
MLS	Velodyne VLP-16	VeloView MATLAB 2020a	RANSAC ASA	LRM CFPD	Apple	Canopy density estimation	[252]
MLS	Stereo vision	ROS	Skeletonization, CNN	TM	Cherry	Pruning	[253]
TLS	FARO Focus S70	PCL 1.11.1 Visual Studio 2022 FV-2200	DTA LA-DT	LRM LAEM	Rapeseed	Leaf estimation	[254]

**Table 6:** Comparison of airborne LiDAR systems (ALSs), terrestrial LiDAR systems (TLSs), and mobile LiDAR systems (MLSs) for crop management in precision agriculture.

Aspect	Airborne LiDAR System (ALS)	Terrestrial LiDAR System (TLS)	Mobile LiDAR System (MLS)
Platform	Aircraft (e.g., UAVs, planes, helicopters)	Stationary tripods or ground vehicles	Moving vehicles (e.g., tractors, ATVs)
Coverage Area	Large-scale, extensive coverage	Small to medium-scale, site-specific	Medium-to-large-scale, linear coverage (e.g., along field rows)
Data Collection	Rapid, wide-area scanning	High-detail, stationary, or limited-area scanning	Continuous, high-density scanning along paths of movement
Altitude/Range	High altitude, large range	Ground level, limited range	Variable altitude, depending on vehicle height and field conditions
Resolution	Moderate to high, depending on altitude and sensor	Very high due to proximity to crops	High, suitable for detailed mapping of crop rows and field features
Applications	Large field surveys, topographic mapping, biomass estimation	Detailed plant structure analysis, canopy height measurement	Field condition monitoring, crop health assessment, yield estimation
Advantages	Extensive coverage, rapid data collection over large areas	High precision and detail, minimal atmospheric interference	Mobility, ability to cover large areas quickly and repeatedly
Limitations	Weather dependent, regulatory restrictions, cost	Limited coverage area, requires multiple setups for large fields	Limited by vehicle access, potential for obstructions in the field
Crop management practices	Field topography, crop biomass, and health mapping	Detailed crop structure analysis, disease detection	Monitoring crop growth, assessing field variability, yield prediction

## Data Availability

All of the data generated or analyzed during this study are included in this published article.

## Nomenclature

ASBS	Alpha Shape by Slices Algorithm
ASA	Alpha Shape Algorithm
APSIM	Agricultural Production Simulator
BEM	Biomass Estimation Model
BPNN	Back Propagation Neural Network
CHM	Crop Height Model
CRM	Crop Row Model
CSM	Canopy Surface Model
CMM	Canopy Metric Model
CSF	Cloth Simulation Filter Algorithm
CFPD	Canopy Foliage Prediction Model
CMA	Canopy Metric Algorithm
CHA	Concave Hull Algorithm
CGA	Computational Geometry Algorithm
CXHA	Convex Hull Algorithm
CVM	Cross Validation Model
DTM	Digital Terrain Model
DEM	Digital Elevation Model
DSM	Digital Surface Model
DTA	Delaunay Triangulation Algorithm
DBSCAN	Density Based Clustering Algorithm
FLASH	Fast Line-of-Sight Atmospheric Analysis of Spectral Hypercubes
GBM	Generalized Boosted Model
GMM	Gaussian Mixture Model
GLM	Generalized Linear Model
GNBA	Gaussian Naïve Bayes Algorithm
IWM	Iterative Waterfall Model
ICPA	Iterative Closest Point Algorithm
IWM	Iterative Waterfall Model
IDW	Inverse Distance Weighting Algorithm
KM	Kinematic Model
KNN	k-Nearest Neighbour Algorithm
LAEM	Leaf Area Estimation Model
LAI	Leaf Area Index
LADE	Leaf Area Density Estimation Model
LA-DT	Leaf Area- Delaunay Triangulation
LBM	Laser Beam Model
QTM	Quantitative Terrain Model
QSM	Quantitative Structure Model
RFR	Random Forest Regression Model
LRM	Linear Regression Model
LLSVM	Lateral Linear Stereoscopic Vision Model
LME	Linear Mixed Effects Model
MCSM	Multitemporal Crop Surface Model
MSM	Multi-Spectral Model
MLRM	Multiple Linear Regression Model
MSM	Maize Segmentation Model
MOSM	Maize Organ Stratification Model
NDVI	Normalized Difference Vegetation Index
OQSM	Optimized Quantitative Structural Model
PLSRM	Partial Least Square Regression Model
PCL	Point Cloud Library
PCA	Poly Cylinder Algorithm
PCSA	Point Cloud Segmentation Algorithm
PCTA	Point Cloud Transformer Algorithm
PCPA	Point Cloud Processing Algorithm
PCAA	Principal Component Analysis Algorithm
PH	Plant Hight
RANSAC	Random Sample Consensus Algorithm
ROS	Robot Operating System
SLAM	Simultaneous Localization and Mapping Algorithm
SVIA	Spectral Vegetation Indices Algorithm
SLMR	Stepwise Multiple Linear Regression
SVMM	Support Vector Machine Model
SDA	Standard Deviation Algorithm
SORA	Statistical Outlier Removal Algorithm
TIN	Triangulation Network Filtering Algorithm
TM	Tree Model
ULR	Univariate Linear Regression
VBA	Voxel-Based Algorithm
3DPI	3D Photo Inpainting
3DPC	3D Point Cloud
3DCM	3D Crop Model
3DLSM	3D Leaf Surface Model
3DSM	3D Surface Model
